# Magnetic Resonance Imaging Measures of Brain Structure to Predict Antidepressant Treatment Outcome in Major Depressive Disorder

**DOI:** 10.1016/j.ebiom.2014.12.002

**Published:** 2014-12-03

**Authors:** Mayuresh S. Korgaonkar, William Rekshan, Evian Gordon, A. John Rush, Leanne M. Williams, Christine Blasey, Stuart M. Grieve

**Affiliations:** a The Brain Dynamics Centre, Westmead Millennium Institute, Sydney Medical School, Sydney, NSW, Australia; b Discipline of Psychiatry, Sydney Medical School, The University of Sydney, Westmead Hospital, Sydney, NSW, Australia; c Brain Resource Ltd, Sydney, NSW, Australia; d Brain Resource Ltd, San Francisco, CA, USA; e Duke-National University of Singapore, Singapore; f Department of Psychiatry and Behavioral Sciences, Stanford University, 401 Quarry Road, Stanford, CA 94305, USA; g Sierra-Pacific Mental Illness Research, Education, Clinical Center (MIRECC), Veterans Affairs Palo Alto Health Care System, Palo Alto, CA 94304, USA; h PGSP-Stanford University Consortium, Palo Alto, CA, USA; i Sydney Translational Imaging Laboratory, Charles Perkins Centre and Sydney Medical School, University of Sydney, NSW 2006, Australia; j Department of Radiology, Royal Prince Alfred Hospital, Camperdown, Sydney, NSW 2006, Australia

**Keywords:** Decision trees, Magnetic resonance imaging, Diffusion tensor imaging, Major depressive disorder, Biomarker predictors, Remission, iSPOT-D, Replication

## Abstract

**Background:**

Less than 50% of patients with Major Depressive Disorder (MDD) reach symptomatic remission with their initial antidepressant medication (ADM). There are currently no objective measures with which to reliably predict which individuals will achieve remission to ADMs.

**Methods:**

157 participants with MDD from the International Study to Predict Optimized Treatment in Depression (iSPOT-D) underwent baseline MRIs and completed eight weeks of treatment with escitalopram, sertraline or venlafaxine-ER. A score at week 8 of 7 or less on the 17 item Hamilton Rating Scale for Depression defined remission. Receiver Operator Characteristics (ROC) analysis using the first 50% participants was performed to define decision trees of baseline MRI volumetric and connectivity (fractional anisotropy) measures that differentiated non-remitters from remitters with maximal sensitivity and specificity. These decision trees were tested for replication in the remaining participants.

**Findings:**

Overall, 35% of all participants achieved remission. ROC analyses identified two decision trees that predicted a high probability of non-remission and that were replicated: 1. Left middle frontal volume < 14 · 8 mL & right angular gyrus volume > 6 · 3 mL identified 55% of non-remitters with 85% accuracy; and 2. Fractional anisotropy values in the left cingulum bundle < 0 · 63, right superior fronto-occipital fasciculus < 0 · 54 and right superior longitudinal fasciculus < 0 · 50 identified 15% of the non-remitters with 84% accuracy. All participants who met criteria for both decision trees were correctly identified as non-remitters.

**Interpretation:**

Pretreatment MRI measures seem to reliably identify a subset of patients who do not remit with a first step medication that includes one of these commonly used medications. Findings are consistent with a neuroanatomical basis for non-remission in depressed patients.

**Funding:**

Brain Resource Ltd is the sponsor for the iSPOT-D study (NCT00693849).

## Highlights

• Our study identified biomarkers which provide clinically actionable information in guiding the prescription of ADMs.
• The MRI protocols used in our study are routinely used clinically, making this biomarker easy to translate to a clinical setting.
• Volumetric measures of the left middle frontal and the right angular gyri can identify a subset of patients who will not remit to three commonly prescribed ADMs.
• Our findings contribute three new objective neuroimaging measures to identify non-remitters prior to initiation of treatment.

## 1 Introduction

Major depressive disorder (MDD) is a chronic disease with a relapsing and remitting course. Antidepressant medications (ADMs) form the front-line treatment for MDD and less than 50% of patients respond or remit to their first treatment (Gartlehner et al., 2012, Hansen et al., 2008). There are currently no objective measures to guide the treatment decisions in MDD, and the clinical standard is to use a “watch and wait” strategy relying on trial and error (Rush et al., 2008). The time taken to conduct iterative trials of different medications represents an enormous source of direct healthcare costs, indirect economic losses and an increase of the total healthcare burden associated with MDD.

Prompted by this context, there has been a recent focus on the development of neurobiological markers (“biomarkers”) including techniques that are able to capture disruptions to the underlying brain circuitry (Insel et al., 2010, Leuchter et al., 2010). These biomarkers are yet to be validated for sufficient clinical utility (Fu et al., 2013, Labermaier et al., 2013). Neuroimaging provides a means to noninvasively capture the spatiotemporal circuitry relationships in the brain that may reflect the functional abnormalities present in depression — hence approaches that use imaging measures of brain abnormalities represent excellent candidates for tests of treatment prediction. Evidence of structural and functional abnormalities in MDD comes from molecular imaging (McGrath et al., 2013), and from multiple MR imaging modalities including diffusion tensor imaging (DTI) (Korgaonkar et al., 2011, Korgaonkar et al., 2012, Korgaonkar et al., 2014a), gray matter (GM) volume from T1 weighted MRI scans (Grieve et al., 2013a), as well as task-based and resting-state functional MRI (Korgaonkar et al., 2013, Greicius et al., 2007). Most studies have concentrated on key circuits thought to be central to the development and maintenance of MDD (e.g., limbic structures including the cingulate cortex and the dorsolateral prefrontal medial orbitofrontal cortices). This approach, however, may limit the power of imaging to capture whole brain patterns of dysfunction.

Also, different imaging measures may capture different aspects of malfunctioning circuits in MDD, and may therefore contribute to treatment prediction in a unique, and likely an independent (and potentially additive) manner. Integrating data across different imaging measures can therefore provide a powerful approach to isolate groups of patients with similar pre-treatment impairments. These groups of patients with common patterns of brain alterations may therefore respond in a similar way to treatments tailored to their underlying circuitry abnormalities. Signal detection analyses employing receiver operator curve (ROC) analysis procedures are well suited to developing dichotomous outcomes from multiple measures (Kraemer, 1992). This analysis assesses different variables at all possible cut points identify an optimal trade-off between sensitivity and specificity.

This report addresses the question of whether pre-treatment brain measures from T1 weighted (volume) and DTI (structural connectivity) MR Imaging sequences can identify individuals who will, or will not, remit during acute phase ADM treatment. Both these imaging sequences are routinely prescribed in clinical neurological assessments and offer an easy translation of findings to a clinical setting. We use signal detection ROC analyses with structural imaging measurements of both GM volume and connectivity across the entire brain, to identify the best possible combination of pre-treatment imaging measures and cut-points to prospectively predict remission status following acute treatment with ADMs. Our aim was to identify general predictors of which patients remit and which patients do not, with the goal of developing a practical algorithm to help inform clinical decision making about ADMs. We tested this aim using data drawn from the imaging sub-study of the International Study to Predict Optimized Treatment in Depression (iSPOT-D). Following the planned design of iSPOT-D, we first evaluated our aims in the test cohort, the first subsample of patients, and then tested for replication in the second validation sub-sample.

## 2 Methods

### 2.1 Participant Characteristics and Study Protocol

Data was gathered from participants in the International Study to Predict Optimized Treatment in Depression (iSPOT-D), for which the study protocol, clinical assessments, inclusion/exclusion criteria and diagnosis procedures have been previously described (Williams et al., 2011, Grieve et al., 2013b). In short, the Mini-International Neuropsychiatric Interview, using DSM-IV criteria, and a 17-item Hamilton Rating Scale for Depression (HRSD_17_) score ≥ 16 confirmed the primary diagnosis of MDD. Participants were not currently suffering or had a history of bipolar disorders, schizophrenia, schizoaffective, psychosis not otherwise specified, anorexia, bulimia, obsessive compulsive disorders or primary post-traumatic stress disorder. All MDD participants were either ADM-naïve or had undergone a wash-out period of at least 5 half-lives of a previously prescribed ADM. Participants were randomized to receive flexibly-dosed, open-label escitalopram, sertraline or venlafaxine-extended release (venlafaxine-ER) for eight weeks. Our study recruited from primary care, community and academic psychiatry settings with the goal of representing a broad sample of antidepressant treatment seekers. Medications were prescribed and doses adjusted by treating clinicians according to routine clinical practice, but following the recommended dose ranges. An HRSD_17_ of ≤ 7 was used to ascribe remission. In addition to the HRSD_17_ score, participant age, gender, age of onset of depression, depression duration, number of previous depression episodes, previous treatment, melancholia, and score of the 42 item depression-anxiety-stress scale were recorded at baseline.

**Fig. 1:**
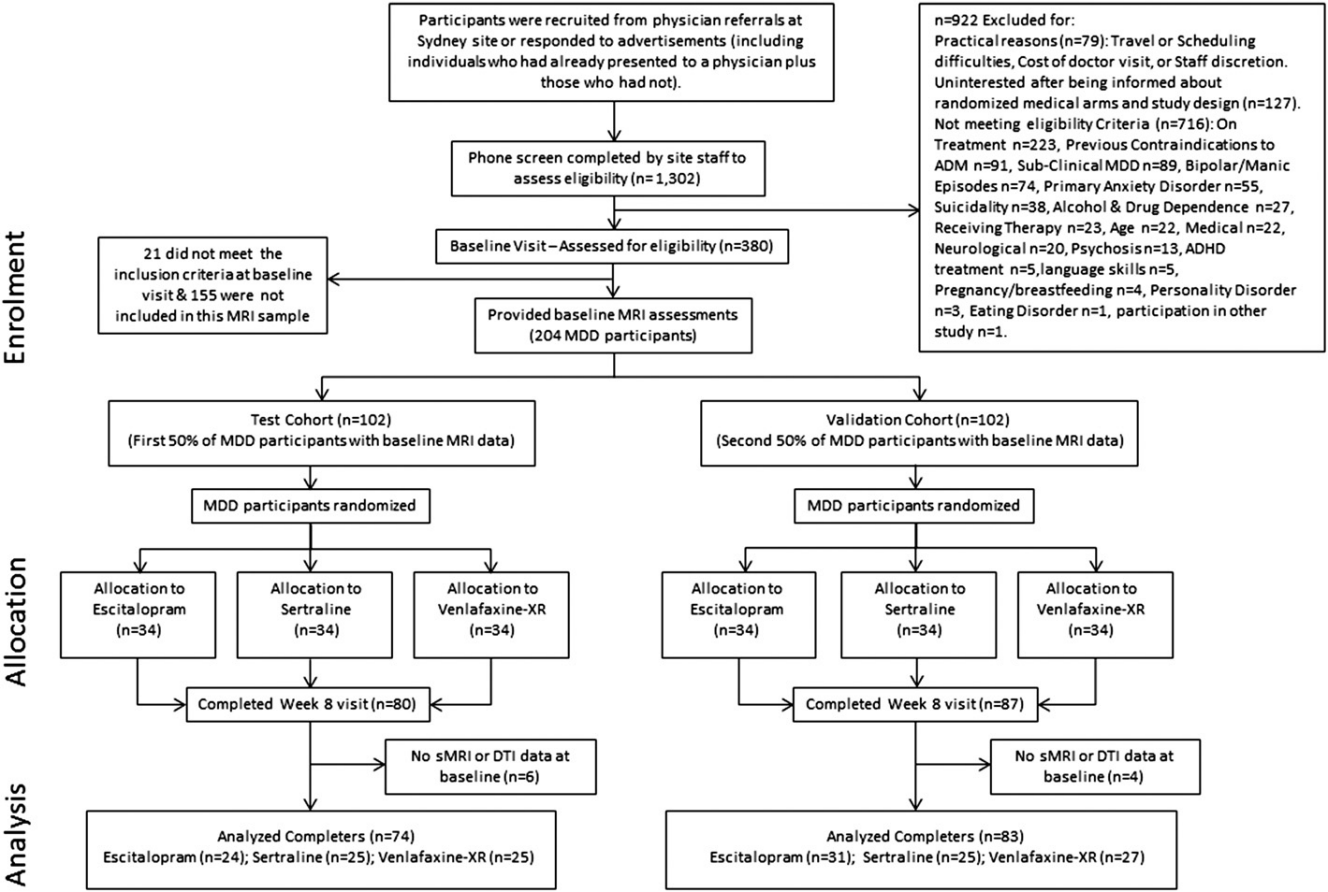
iSPOT-D study CONSORT Diagram. Abbreviations: ADMs, antidepressant medications; DTI, diffusion tensor imaging; MDD, major depressive disorder; sMRI, structural T1 MRI.

As per the analysis plan, the first 50% of the MDD participants who completed imaging at baseline visit were used as the test cohort (n = 102) and the second 50% of the MDD participants as the validation cohort (n = 102) (Grieve et al., 2013b). Fig. 1 provides the CONSORT diagram. 80 and 87 participants completed their 8-week course of assigned ADM in the test and validation cohorts respectively. Of the 80 participants from the test cohort, six participants did not complete the DTI scan while four participants did not complete the T1 structural scan resulting in 74 and 76 participants for each analysis. For the validation cohort, DTI and T1 data from 83 participants who completed the clinical follow-up at week 8 were available for analysis. These sample sizes represent the biggest cohort to be used to identify imaging prognostic markers for ADM treatment. Based on effect sizes from previous work in the field, we anticipated these sample sizes to provide sufficient power for analysis. The Western Sydney Ethics Committee approved this study and all participants provided written informed consent.

### 2.2 Image Acquisition and Analysis

DTI and T1-weighted sagittal 3D SPGR MRI data were acquired using a 3 Tesla GE Signa HDx scanner (GE Healthcare, Milwaukee, Wisconsin) as previously described (Grieve et al., 2013b). Volumetric analysis was performed using voxel-based morphometry (VBM8), and 116 cortical and subcortical brain regions were generated using the Automated Anatomical Labeling (AAL) atlas (Grieve et al., 2013a, Tzourio-Mazoyer et al., 2002). DTI data analyzed using Tract-Based Spatial Statistical analysis (TBSS) to generate fractional anisotropy (FA) measurements for 46 major white matter tracts in the brain using the Johns Hopkins University International Consortium for Brain Mapping (JHU ICBM)-DTI-81 white matter labels atlas (Korgaonkar et al., 2011, Mori et al., 2008). Details for the MRI sequences and volume and DTI analyses are provided in the supplementary section.

### 2.3 Statistical Analyses

ROC analyses, based on signal detection methods (Kraemer, 1992) were used to identify which MRI measures (GM region/white matter tract), and at what level (volume or FA), optimally discriminate non-remitters and remitters. This analysis is non-parametric and operates via a recursive partitioning procedure. This approach is designed to handle multiple variables and as compared to traditional regression analysis methods can analyze all possible interactions, rather than only those specified a priori and can analyze interactions even when the main effects are not included in the model. More specifically, for each measured potential predictor, cutoff points are generated at all values observed in the variable. The quality of a cutoff point is based on its ability to divide the sample into 2 subsamples maximally distinct in discriminating non-remitters and remitters. A kappa statistic is calculated for each cut-point, and the largest kappa coefficients correspond to cut-points with maximum sensitivity and specificity (Kraemer, 1992) (QROC available at mirecc.stanford.edu).

**Fig. 2:**
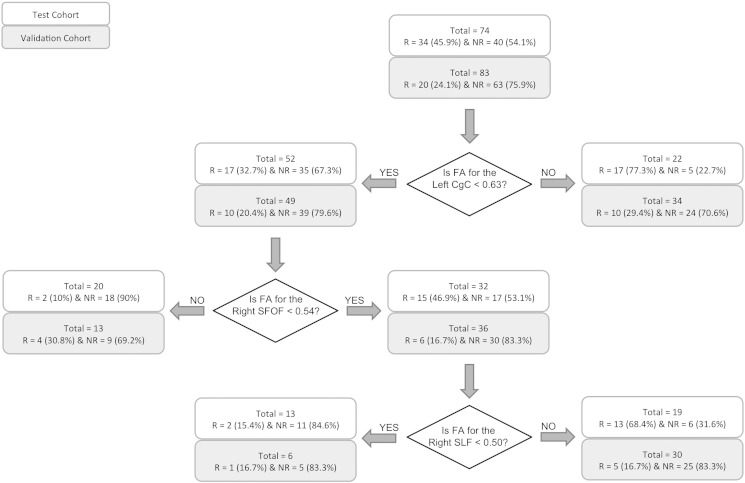
Decision tree for prediction of remission using the DTI measures. Abbreviations: R, remitters; NR, non-remitters; FA, fractional anisotropy; CgC, cingulum portion of the cingulate gyrus; SFOF, superior fronto-occipital fasciculus; SLF, superior longitudinal fasciculus.

**Fig. 3:**
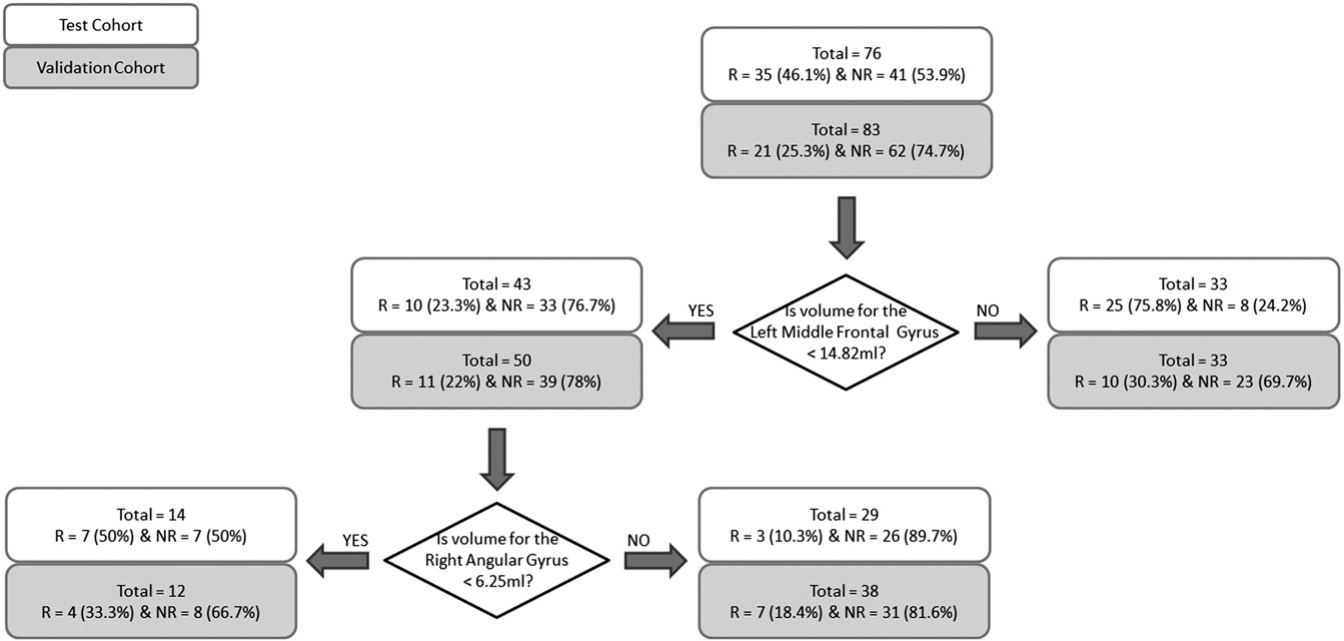
Decision tree for prediction of remission using the volumetric measures. Abbreviations: R, remitters; NR, non-remitters.

The cutoff point that yields the best prediction is identified across all values of all variables. That cutoff point is then used to divide the total sample into 2 subsamples. The same procedure is repeated systematically in each of the 2 subsamples. This iterative process continues until failure to reach a significance group difference at p < 0.01 for any candidate cutoff value. After the last step of the ROC analysis is reached, the probability of remission for each subgroup is calculated and the results presented as hierarchical decision tree diagrams (as in Fig. 2, Fig. 3).

Three separate ROC analyses were conducted: 1. Using volumetric measures for all 116 cortical–subcortical brain regions, 2. Using FA measures for all 46 white matter tracts, 3. Using all volumetric, FA, demographic and clinical measures. For each analysis, optimal cutoff points for prediction of remission status were first identified using the test cohort, following this, the performance of these cutoff values was assessed in the validation cohort using the binomial test. We also tested if these cutoff points performed better than chance (i.e. better than a remission rate of 35% of patients based on existing literature and on remission rates observed in the overall iSPOT-D study cohort, n = 1700 at time of analysis) (Thase et al., 2001, Rush et al., 2006). The proportion of non-remitters in the validation cohort was much higher than that observed in the test cohort and previously published prevalence rate for ADM use (Table 1). To remove this bias in testing the validity of the decision trees, an additional cross-validation procedure was performed using 1000 iterations of 100 MDD participants randomly chosen across both cohorts. This approach would also provide the confidence interval for the classification accuracy for the identified cutoffs. The limited sample size of remitters vs. non-remitters after splitting by treatment arms precluded us from testing treatment arm effects in our analysis.

## 3 Results

### 3.1 Participant Characteristics

Table 1 shows the clinical and demographic characteristics of the test (n = 74) and validation (n = 83) cohorts overall and for each group subdivided by remission status at 8 weeks. The average daily doses (mg/day) (± S.D.) at week 8 for the treatment arms were: escitalopram = 13 ± 5; sertraline = 61 ± 27; and venlafaxine-ER = 100 ± 35. Remission rates within each cohort were similar across treatment arms. Significant cohort differences existed for baseline and week 8 symptom severity (HRSD_17_ Baseline, HRSD_17_ Week 8: validation cohort > test cohort; p < 0 · 05), however, improvement in symptoms (HRSD_17_% change), age of onset and duration of illness was similar for both cohorts. The remission rates were lower in the validation cohort: 46% (34/74) of the MDD participants from the test cohort achieved remission, while 24% (20/83) of the MDD participants from the validation cohort achieved remission (*χ*^2^ = 8 · 02; p = 0 · 005).

### 3.2 Identification of Optimal Cutoffs for Remission Status Using the Test Cohort

Fig. 2 shows the decision tree identified using the DTI measures alone. The ROC model identified three key cutoff points (in order):

(1) FA = 0 · 63 for the left cingulum portion of the cingulum bundle (L-CgC), where 77% of patients greater or equal to this value were remitters (17/22; with no further significant decision points applying to this group) and 67% of patients less than this value were non-remitters (35/52).
(2) FA = 0 · 54 for the right superior fronto-occipital fasciculus (R-SFOF) (threshold applied to the L-CgC < 0 · 63 group), where 90% of patients greater or equal to this value were non-remitters (18/20; no further decision points applying to this group); and 53% of subjects less than this value were non-remitters (17/32).
(3) FA = 0 · 50 for the right superior longitudinal fasciculus (R-SLF) (threshold applied to the R-SFOF < 0 · 54 group), where 85% of the patients less than this value were non-remitters (11/13).

Based on this analysis, there were two decision paths which had sufficient classification accuracy (> 80%) for clinical action i.e. to identify a meaningful proportion of depressed patients who were unlikely to remit acutely to medication: 1. Participants with L-CgC < 0 · 63 & R-SFOF > 0 · 54; and 2. Participants with L-CgC < 0 · 63, R-SFOF < 0 · 54 & R-SLF < 0 · 50.

Fig. 3 shows the decision tree identified using the volumetric measures alone. The decision tree for the combined measures model i.e. FA, volume, clinical and demographic measures was *identical* to the volumetric decision tree i.e., only volumetric parameters were significant contributors and no DTI or characteristic parameters were selected as critical decision points. Two key cutoff points were identified in this model (in order):

(1) Volume = 14 · 82 ml for the left middle frontal gyrus, where 76% of patients greater or equal than this value were remitters (25/33; with no further significant decision points applying to this group) and 77% of patients less than this value were non-remitters (33/43).
(2) Volume = 6 · 25 ml for the right angular gyrus (threshold applied to the left middle frontal < 14 · 82 ml group), where 50% of patients less than this value were remitters (7/14); while 90% of patients greater than or equal to this value were non-remitters (26/29).

From this analysis, participants with the left middle frontal < 14 · 82 & right angular gyrus volume > 6 · 25 were most unlikely to remit.

### 3.3 Performance of Identified Cut Points in the Validation Cohort & Relative to Chance

The performance of the identified decision trees in the validation cohort is shown in Fig. 2, Fig. 3 (gray boxes marked as validation cohort at each cut point).

Of the two decision tree cut-offs identified using the FA measures, only one was found to replicate in accuracy. Accuracy of identification of non-remission in patients with L-CgC < 0 · 63 & R-SFOF > 0 · 54 was 69% in the validation cohort (i.e. 9/13 and significantly different at p = 0 · 034 as compared to 90% for the test cohort using the binomial test); while that for patients with L-CgC < 0 · 63, R-SFOF < 0 · 54 & R-SLF < 0 · 50 replicated at 83% (5/6 and as compared to 85% for the test cohort; p > 0 · 05 using the binomial test).

The decision tree cut-offs identified using the volume measures i.e. left middle frontal volume < 14 · 82 ml & right angular gyrus volume > 6 · 25 ml for higher probability for non-remission, accurately predicted non-remission in 82% of patients (i.e. 31/38) and were not significantly different in identification accuracy as compared to that from the test cohort (90% or 26/29 for the test cohort; p > 0 · 05 using the binomial test).

Of the two replicated decision trees, the volumetric measures decision tree identified a more meaningful number of patients who did not remit (55% of all non-remitters pooled across the Test and validation cohorts i.e. 57/103), compared to the DTI-based decision tree which identified only 15% of all non-remitters (i.e. 16/103). This decision tree also performed significantly better than chance in identifying non-remission status for the test (p = 0 · 002), validation (p = 0 · 019) and also for the pooled cohort (p < 0 · 001).

### 3.4 Cross Validation Analysis of the Volumetric Decision Tree Using the Pooled (test and validation) MDD cohort

The remission rates were lower in the replication cohort (*χ*2 = 8.02; p = 0.005): 46% (34/74) of the MDD participants from the test cohort achieved remission, while 24% (20/83) of the MDD participants from the replication cohort achieved remission. To remove any bias of this high proportion of non-remitters in the replication cohort on the predictive accuracy in this cohort and to provide a distribution and confidence interval of this classification accuracy, a multiple sampling bootstrap analysis procedure was employed. This cross-validation procedure was performed on MDD participants pooled across the test and replication cohorts and was tested for the volumetric decision tree that identified the largest number of non-remitters. One thousand random groups with 100 MDD participants randomly chosen from the pooled cohorts each time in each group were analyzed for classification of non-remitters using the volumetric decision tree. The mean specificity from this analysis was 85.0 ± 5.7% and was found to match that obtained from the analysis above suggesting that our findings were not biased due to the incidental low remission rate in the validation cohort. The distribution of percentage of non-remitters correctly identified was 51.7 ± 6.3%.

### 3.5 Description of Non-remitted Patients Identified Using the Decision Tree

The decision tree based on volumetric measurements identified a significant cohort of all non-remitters; we therefore characterized the demographic and clinical features of this selected non-remitter (S-NR) sample using the pooled sample. We compared the demographic and clinical characteristics of this group to the non-selected non-remitters (N-NR) and to the rest of the group (i.e. all remitters + N-NR) using independent t-tests or chi-squared tests (Table 2).

The only significant difference between the S-NR and N-NR was in the proportion of melancholics (*χ*^2^ = 4 · 27; p = 0 · 039; 89% participants were non-melancholics in S-NR vs. 74% in N-NR). Compared to the rest of the group, the S-NR were significantly older, had been previously treated for depression, exhibited greater frequency and intensity of side-effects, were severe at week 8 (HRSD_17_) and showed less improvement in severity (as measured by % reduction in HRSD_17_), however none of these characteristics were selected as contributors to an effective decision tree in the ROC analysis. The S-NR group did not significantly differ from the rest of the cohort in relation to prescribed treatment type or dose.

### 3.6 Overlap Between DTI and GM Volumetric Selected Non-remitter Groups

To further explore the groups of patients selected using the DTI and the structural volume measurements, we first examined the degree of overlap between the S-NR using each decision tree. When these two decision trees are applied in series (i.e., first applying the DTI, then the volumetric decision tree — or vice versa) to the pooled sample, no remitters and 10 non-remitters were selected (i.e., the test was 100% accurate in classifying non-remitters for the selected MDD group). When the same decision trees are applied in parallel (i.e. including all MDD participants who were selected by applying either the DTI or the volumetric decision trees), 13 remitters and 63 non-remitters were selected (i.e. the test was 83% accurate in classifying non-remitters for the selected MDD group).

### 3.7 Voxel Based Morphometry Analysis of Volume Data

**Fig. 4:**
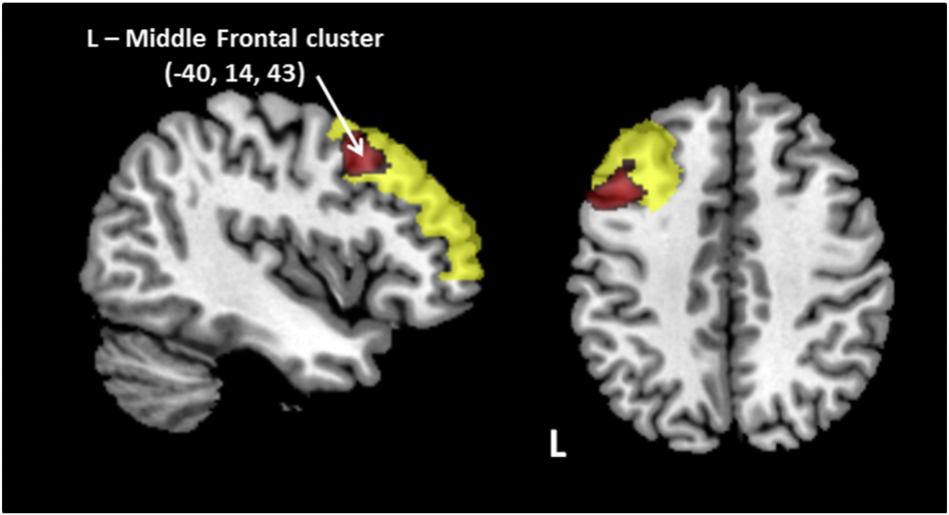
Significant left middle frontal cluster (shown in red) identified in the whole brain VBM analysis comparing selected non-remitters identified from the volumetric decision tree & all remitted MDD participants. The cluster peak was at (− 40, 14, 43) and comprised of 700 voxels (p < 0.05 FWE corrected). The whole middle frontal gyrus is shown in yellow. Abbreviations: L, left.

We performed voxel based morphometry analyses to refine the anatomical location of the left middle frontal and right angular gyrus GM predictive regions, and to identify any additional areas of the brain associated with remission. Two sets of comparisons were performed: 1. A whole brain analysis comparing S-NR and all remitted patients; and 2. A whole brain analysis comparing all non-remitter and all remitted patients. Only a cluster in the left middle frontal gyrus was found to be significant at the whole brain level (p < 0 · 05 FWE) for the S-NR vs. all remitted patients (S-NR with reduced volume), adding anatomical specificity to the findings from the ROC analysis. The cluster represented 6% of the total ROI and is shown in Fig. 4. No significant whole brain clusters were found for the comparison of all non-remitters and all remitted patients. We next tested if the accuracy in predicting non-remission using volume for the refined left middle frontal cluster improved relative to using volume for the whole region. The peak NR prediction accuracy using this measure was 89% (with 38/103 NR selected). When used in place of the larger left middle frontal gyrus ROI, the GM volume decision tree accuracy improved to 90% (applied to 44/103 NR).

## 4 Discussion

This study found that magnetic resonance imaging measures of brain structure and connectivity acquired pre-treatment could provide clinically actionable information about which patients were unlikely to achieve remission, versus those likely to remit, following acute treatment with three commonly used ADMs. We found that volumetric measures of the left middle frontal and the right angular gyri could reliably identify a subset of patients who did not remit to any of the three prescribed ADMs. Thresholds identified using these measures predicted non-remission status with an accuracy of 82% and selected 55% of all non-remitters in the cohort. The findings contribute new knowledge about objective neuroimaging measures for identifying a large proportion of non-remitters before treatment begins, going beyond the current knowledge on clinical predictors of non-remission (Rush et al., 2008, Labermaier et al., 2013). Pre-treatment measures that predict non-efficacy with a substantial degree of precision to certain treatments can enable ruling out of these treatments and earlier initiation of finding alternative treatment options.

Neuroimaging techniques have shown promise in the identification of neurobiological substrates underlying major depressive disorder and other psychiatric illnesses. The limbic-cortical pathways have been identified as the key brain network that may guide treatment for depression (Fu et al., 2013, Mayberg, 2003, Korgaonkar et al., 2014b). A number of baseline MRI measures of both function and structure of these regions have been associated with treatment remission. Fu et al., 2013 It seems likely, however that more than one tract or brain region may be required for effective treatment prediction. Association analyses are helpful to identify candidate biomarkers; however to employ such a biomarker in a clinical setting would need a demonstrated reliable predictive power along with decision points applicable at an individual level. A major challenge in the field, therefore, is to find ways of combining different brain measures to generate an integrated tool to base clinical treatment decisions on. Data driven classification techniques such as pattern classification are promising in combining whole brain neuroimaging measures to get objective information on diagnosis or prognosis (Fu et al., 2008). This approach has successfully been applied using volumetric data to distinguish between unipolar and bipolar depression (Redlich et al., 2014). The framework of the ROC based signal detection analysis utilized in this paper provides a non-biased and completely data driven method to identify regions and cut-points for categorization of treatment outcome. In our analysis, this approach was robust in identifying non-remitters and was replicated in an independent cohort. However, we do note that the cut-points identified at each partitioning stage using the test cohort were not replicated as significant discriminators using the Fisher's exact statistic in the validation cohort. We think this is likely due to the differential non-remission rate for the validation cohort. Our validation data does show robust replication of the main goal of the study which was to identify a sub-group of patients for which a combination of measures predicts non-remission with high classification accuracy. This was also supported by our multiple sampling cross-validation analysis.

The decision tree based on the regions and their cut-points identified in our approach provides a measure of classification accuracy which is the probability of either remission or non-remission to ADMs. Defining an overall sensitivity/specificity in the context of our analysis is complex and not clinically meaningful, since we are aiming to isolate groups of subjects that can be classified with a high degree of accuracy, a process that may leave a proportion of the group who are, from a clinical perspective, essentially “unclassified”. For example, based on the DTI decision tree (see Fig. 2), patients for whom left CgC < 0.63 & right SFOF > 0.54 & right SLF > 0.5 cannot be reliably classified. In these cases the decision tree and test will not have helped, and usual clinical care would proceed. An analogous situation of such an approach is the application of an altered management pathway for BRAC1/2 + patients in screening for breast cancer. For this reason we have carefully reported the raw numbers of patients (to allow for any transparent post hoc analysis), and have preferred to refer to accuracy of classification as defined above.

Major depressive disorder is highly heterogeneous, which provides a significant challenge in the management of this disorder (Goldberg, 2011). This heterogeneity was reflected in our analysis, where only a subset of non-remitters was identified using MRI measures. The selected non-remitters were not demographically or clinically different from the non-selected non-remitters except for the marginally lower proportion of those with melancholic features present in the group. Our data supports the use of neurobiological markers to characterize in meaningful ways the heterogeneity in depression not adequately captured using clinical measures (Insel et al., 2010). It would be of interest to further characterize this group of patients using other biological measures (including other forms of imaging, neurophysiological measures, and genetics). In particular, multimodal imaging, combining functional and structural measures offer the most logical approach to further extend out understanding of the features that govern treatment outcomes in this complex disorder (Teipel et al., 2014). Functional data using resting state fMRI (Li et al., 2013), and metabolic information (McGrath et al., 2013), would be expected to add valuable independent information to a treatment-prediction approach as illustrated by our current analysis.

Our voxel based morphometry analysis of the S-NR group provided a more specific cluster of reduced volume within the left middle frontal gyrus — which improved the specificity of this step of the decision tree from 77% to 89%. At a whole brain level no other GM regions were associated with this S-NR group. The significance of this specific structural feature in the selected- non-remitter group was highlighted by a failure to identify any significant GM regions when a comparison was performed comparing remitters with non-remitters in the whole cohort. The significant cluster corresponds to the dorsolateral prefrontal cortex, a region implicated in a number depression studies, which is widely held to be involved in the control of executive and emotion functions in the brain (Hamilton et al., 2012, Fitzgerald et al., 2008). Our previous analysis of GM volume reduction at baseline in MDD demonstrated profound GM reductions in this region compared to normal subjects (Grieve et al., 2013a). Our findings also extend validation of reduced GM volume in this region found to be associated with reduced likelihood of antidepressant response in a recent meta-analysis (Fu et al., 2013). The right angular gyrus was the second component of our optimal decision tree, the exact role of this region in depression is poorly understood, but may relate to its putative involvement in the default mode network, cognition (e.g. memory, sematic processing, attention) or in its role in the synthesis of complex social and environmental information (Seghier, 2013).

While the GM volumetric decision tree was superior to the DTI tree, the latter model was still significant and produced comparable overall results (84% accuracy). More importantly, the DTI decision tree had considerable overlap with the GM volumetric tree with 100% accuracy of non-remission in individuals (n = 10) who were identified by both tests as non-remitters. Although the fraction of subjects both these tests applied to is small, this result does illustrate the potential of additive (in series or in parallel) usage of such data in a clinical algorithm. A great deal of literature highlights functional brain changes as predictors of treatment outcome in MDD (Fu et al., 2013, Mayberg et al., 1997). The inclusion of fMRI measures may add further value to the predictive algorithms we have identified.

This study had several limitations. The iSPOT-D trial is a naturalistic study, and as such, lacks a placebo arm, this places important limitations on how we can use this data to understand the brain circuits that govern remission — the most critical of these being differentiating the specific effects of ADM therapy from spontaneous remission. On the other hand, the design of the iSPOT-D trial to mimic routine clinical practice means that our results reflect a real world setting. We included both participants who were ADM naïve or had previous history of ADM use. Although participants with previous ADM use underwent a washout period prior to participating in the study, the long-term impacts of ADM use on brain structural features that may impact on remission are not well understood. It is also possible that participants with previous ADM use may represent a treatment resistant group. As a result, the sensitivity of our findings with respect to medication history should be inferred with caution. Our findings are limited to the three commonly prescribed ADMs used in the study, the generalizability of these findings to other classes of ADMs currently available needs further work. Although our results provide encouraging evidence that neuroimaging measures can play a role in *ruling out* ADM use, markers that may reliably predict treatment success for a particular patient still remain to be identified. An inherent limitation of the employed approach is that the test could be applied only in MDD participants who met cutoff criteria for the identified measures. This may lower the overall sensitivity and specificity of prediction when considering the whole cohort. However, a test, albeit applicable to a smaller group but which has a greater reliability in identifying whether a patient will not remit to ADMs, would still be clinically meaningful and an improvement to current practice. Further work is required to understand the role that neuroimaging may play in guiding the use of other types of depression treatments (e.g. cognitive behavioral therapy, repetitive transcranial magnetic stimulation, deep brain stimulation, etc.) versus ADMs or combination treatment regimes.

These limitations notwithstanding, this report shows that measures derived from routinely prescribed clinical MRI scans have the potential to inform decision to not prescribe antidepressants in patients with major depressive disorder. The goal of our study was to identify an algorithm that could be easily employed in current clinical practice. Structural volumetric T1 weighted and DTI MRI scans are routinely clinically prescribed for neurological evaluations and a biomarker based on these measures would be easy to employ in a clinical setting. Our analysis also used standard anatomical atlases — ensuring reproducibility and standardization of our findings. These data contribute towards a neuroanatomical basis for non-remission in depressed patients.

## Financial Disclosures

MSK has no disclosures to declare.

WR is an employee of Brain Resource Ltd and has stock options in the company.

EG is the CEO of Brain Resource Ltd and has significant equity and stock options in the company.

AJR has received consulting fees from Brain Resource Ltd, H. Lundbeck A/S, National Institute of Drug Abuse, Eli Lilly, and Medavante, Inc; speaking honoraria from Otsuka, University of California, San Diego, New York State Psychiatric Institute, The American Society of Clinical Psychopharmacology and Hershey Medical Centre; royalties from Guilford Publications, a travel grant from CINP and research support from Duke-National University of Singapore.

LMW has received fees as a consultant for Brain Resource Ltd and did hold stock in Brain Resource Ltd.

CB has received fees as a consultant for Brain Resource Ltd.

SMG has received fees as a consultant for Brain Resource Ltd.

## Author Contributions

SMG and LMW oversaw the structural imaging design for iSPOT-D. MSK, EG, AJR, SMG and LMW conceived the idea of applying the ROC analysis methodology to imaging data. MSK & SMG performed the literature search, imaging data analyses, statistical analysis, and wrote the first draft of the manuscript. CB provided expertise on the implementation and interpretation of the ROC analyses. WR provided statistical advice and performed ROC analysis of the test cohort. EG & AJR provided advice on the clinical interpretation of findings. All authors provided major edits and advice in the write up of manuscript. LMW was the academic principal investigator for iSPOT-D 2008–2013, co-PI for the Sydney site, and led the design of the overall iSPOT-D study. All authors have approved the final submitted version.

## Role of Funding Source

BRC Operations Pty Ltd was the sponsor for the iSPOT-D study. The sponsor played a role in trial design and data collection. The sponsor had no role in the current study design, analysis or interpretation, or in the writing of this report.

## Figures and Tables

**Table 1:** Demographics and clinical measures summary.

	Test cohort						Validation cohort					
Characteristics	All		Remission				All		Remission			
			Yes		No				Yes		No	
	N	%	N	%	N	%	N	%	N	%	N	%
Number	74	100	34	46	40	54	83	100	20	24 · 1	63	75 · 9
No. of Females	37	50	16	47	21	53	42	51	11	55	31	49

	Mean	SD	Mean	SD	Mean	SD	Mean	SD	Mean	SD	Mean	SD

Age (years) a	33 · 0	12 · 7	28 · 2	7 · 4	37 · 1	14 · 8	35 · 5	11 · 2	33 · 2	10 · 5	36 · 3	11 · 4
HRSD_17_ Baseline^c^	20 · 7	3 · 6	21 · 2	3 · 8	20 · 3	3 · 4	22 · 3	3 · 4	21 · 2	3 · 3	22 · 7	3 · 4
HRSD_17_ Week 8^a^, ^b^, ^c^	9 · 0	5 · 0	4 · 6	1 · 9	12 · 8	3 · 5	11 · 3	4 · 8	5 · 2	1 · 3	13 · 2	3 · 8
HRSD_17_% change^a^, ^b^	55 · 1	27 · 2	78 · 2	9 · 4	35 · 6	21 · 2	49 · 2	21 · 0	75 · 2	6 · 2	41 · 0	16 · 8
Age of onset (years)	20 · 2	11 · 1	18 · 9	7 · 7	21 · 4	13 · 4	22 · 4	8 · 5	20 · 6	9 · 2	23 · 0	8 · 3
Disease duration (years)^a^	12 · 2	12 · 0	8 · 8	6 · 5	15 · 2	14 · 7	12 · 6	11 · 0	12 · 1	12 · 1	12 · 8	10 · 8

Abbreviations: HRSD_17_, 17-item Hamilton Rating Scale for Depression; SD, Standard deviation.

a Difference between remitters and non-remitters at p < 0 · 05 for test cohort.

b Difference between remitters and non-remitters at p < 0 · 05 for validation cohort.

c Difference between the test and validation cohorts at p < 0 · 05.

**Table 2:** Characteristics of selected non-remitting participants compared to non-selected subject groups.

Characteristics	Selected NR		Non-selected NR			All non-selected (non-selected NR + all remitters)		
	(n = 57)		(n = 46)			(n = 102)		
n in Rx arm (E/S/V)	23/14/20		16/18/12		NS	34/38/30		NS

	N	%	N	%		N	%	

Females	27	47 · 4	25	54 · 3	NS	52	51 · 0	NS
Melancholic⁎	6	10 · 5	12	26 · 1	0 · 039	23	22 · 5	NS
Previous treatment	17	30 · 4	17	40 · 0	NS	53	52 · 0	0 · 009

	Mean	SD	Mean	SD		Mean	SD	

Age	37 · 7	12 · 2	35 · 1	13 · 2	NS	32 · 1	11 · 3	0 · 004
Rx dose (mg/day)	63 · 2	51 · 5	54 · 6	41 · 7	NS	52 · 5	38 · 6	NS
HRSD_17_ Baseline	21 · 4	3 · 6	22 · 5	3 · 5	NS	21 · 9	3 · 6	NS
HRSD_17_ Week 8	12 · 8	3 · 8	13 · 4	3 · 6	NS	8 · 7	5 · 1	0 · 000
HRSD_17_% change	39 · 6	16 · 8	38 · 8	19 · 5	NS	59 · 8	23 · 9	0 · 000
Age of onset	23 · 3	11 · 4	21 · 8	9 · 4	NS	20 · 6	8 · 8	NS
Disease duration	14 · 0	10 · 0	12 · 8	14 · 2	NS	11 · 0	11 · 6	NS
HRSD_17_ anxiety	7 · 0	2 · 1	7 · 5	1 · 9	NS	7 · 1	1 · 8	NS
HRSD_17_ non-anxiety	14 · 4	2 · 6	15 · 0	2 · 5	NS	14 · 8	2 · 7	NS
FIBSER frequency	1.74	1 · 32	1 · 46	1 · 39	NS	1 · 05	1 · 32	0 · 002
FIBSER intensity	1 · 65	1 · 17	1 · 54	1 · 26	NS	1 · 12	1 · 20	0 · 008
FIBSER burden	1 · 04	1 · 05	1 · 02	1 · 20	NS	0 · 73	1 · 03	NS

Abbreviations: E, escitalopram; FIBSER; Frequency, Intensity and Burden of Side Effects Rating; HRSD17, 17-item Hamilton Rating Scale for Depression; NR, Non-remitters; NS, Not significant; Rx, Medication; SD, Standard deviation; S, sertraline; V, venlafaxine-extended release.

⁎ Melancholia was defined based on a score > 7 on the Clinical Outcome in Routine Evaluation scale (Korgaonkar et al., 2011).
