# Supplementary material for: Magnetic Resonance Imaging Measures of Brain Structure to Predict Antidepressant Treatment Outcome in Major Depressive Disorder
Source: eBioMedicine. 2014 Dec 3;2(1):37–45. doi: 10.1016/j.ebiom.2014.12.002 (PMC4484820; doi:10.1016/j.ebiom.2014.12.002)

**SUPPLEMENTARY SECTION**

***MRI Acquisition and Analysis Details***

***Image acquisition***

Magnetic Resonance Images were acquired using a 3·0 Tesla GE Signa HDx scanner (GE Healthcare, Milwaukee, Wisconsin). Acquisition was performed using an 8-channel head coil. T1-weighted sagittal 3D SPGR images were obtained with an isotropic resolution of 1mm (TR=8·3 ms; TE=3·2 ms; Flip Angle=11°; TI=500 ms; NEX=1; ASSET=1.5; Frequency direction: S/I; 180 slices; 1mm slice thickness; 256x256 matrix). Diffusion tensor images were acquired using a spin-echo DTI-Echo Planar Imaging sequence. Seventy contiguous 2·5 mm slices were acquired in an axial orientation with an in-plane resolution of 1·72 mm x 1·72 mm and a 128 x 128 matrix (TR: 17000 ms; TE: 95 ms; Fat Saturation: ON; NEX: 1; Frequency direction: R/L). A baseline image (b=0) and 42 different diffusion orientations were acquired with a b-value of 1250. Total acquisition time for the DTI protocol was 13min 36s.

***Voxel-based morphometry analysis of T1 data***

T1 image data was pre-processed and analyzed using the VBM8 toolbox (http://dbm.neuro.uni-jena.de/vbm.html) and the SPM8 software package (<http://www.fil.ion.ucl.ac.uk/spm>) details of which have been previously described.^1^ Images were corrected for bias-field inhomogeneity; tissue-classified into GM, white matter and cerebrospinal fluid; and registered to standard MNI space using high-dimensional DARTEL normalization.^2^ This approach uses both an affine and a non-linear deformation for normalization using the 550 healthy subject DARTEL MNI template provided as part of VBM8 software. The segmentation approach is based on an adaptive maximum a posterior technique which does not need a priori information about tissue probabilities. The segmentation procedure is further refined by accounting for partial volume effects and by applying a hidden Markov random field model which incorporates spatial prior information of the adjacent voxels into the segmentation estimation. The warped tissue type images were modulated to preserve the volume of a particular tissue within a voxel by multiplying voxel values in the segmented images by the non-linear determinants derived from the spatial normalization step. The analysis of these modulated images allows testing for regional differences in absolute volume of tissue class corrected for individual brain sizes. Finally, images were smoothed with a full-width half-maximum kernel of 8 mm. The Automated Anatomical Labeling (AAL) atlas was used extract volume for 116 cortical and subcortical brain regions (Supplementary Figure).^3^ In addition, volume for the total brain, grey matter, white matter and cerebrospinal was also estimated and included in the analysis.

***Tract-based spatial statistical analysis of DTI data***

DTI data processing and analytic methods have been described in detail. ^4, 5^ DTI data was preprocessed and analyzed using the Oxford Centre for Functional MRI of the Brain (FMRIB) Diffusion Toolbox and Tract-Based Spatial Statistical analysis (TBSS) software tools as part of the FMRIB Software Library release 4·1·3 (<http://www.fmrib.ox.ac.uk/fsl>).^6^ Diffusion tensor models were fitted and images of FA were generated for each participant. An average FA image was generated and thinned to create a white matter skeleton representing the centers of all white matter tracts common to all participants. This FA skeleton was then thresholded to FA ≥0·2 to include the major white matter pathways but avoid peripheral tracts. The Johns Hopkins University International Consortium for Brain Mapping (JHU ICBM)-DTI-81 white matter labels atlas was used to identify parts of the tract skeleton for the 46 major white matter tracts in the brain (Supplementary Figure).^7^ The mean FA for each tract was calculated and used for further analyses.

**References:**

1. Grieve SM, Korgaonkar MS, Koslow SH, Gordon E, Williams LM. Widespread reductions in gray matter volume in depression. NeuroImage Clinical 2013; 3: 332-9.

2. Ashburner J. A fast diffeomorphic image registration algorithm. NeuroImage 2007; 38(1): 95-113.

3. Tzourio-Mazoyer N, Landeau B, Papathanassiou D, Crivello F, Etard O, Delcroix N, et al. Automated anatomical labeling of activations in SPM using a macroscopic anatomical parcellation of the MNI MRI single-subject brain. NeuroImage 2002; 15: 273-89.

4. Korgaonkar MS, Cooper NJ, Williams LM, Grieve SM. Mapping inter-regional connectivity of the entire cortex to characterize major depressive disorder: a whole-brain diffusion tensor imaging tractography study. Neuroreport 2012: 566-71.

5. Korgaonkar MS, Grieve SM, Koslow SH, Gabrieli JDE, Gordon E, Williams LM. Loss of white matter integrity in major depressive disorder: evidence using tract-based spatial statistical analysis of diffusion tensor imaging. Human brain mapping 2011; 32: 2161-71.

6. Smith SM, Jenkinson M, Johansen-Berg H, Rueckert D, Nichols TE, Mackay CE, et al. Tract-based spatial statistics: voxelwise analysis of multi-subject diffusion data. NeuroImage 2006; 31: 1487-505.

7. Mori S, Oishi K, Jiang H, Jiang L, Li X, Akhter K, et al. Stereotaxic white matter atlas based on diffusion tensor imaging in an ICBM template. NeuroImage 2008; 40(2): 570-82.

**Supplementary Figure: Grey matter regions (left) and white matter tracts (right) of the brain from the Automated Anatomical Labeling (AAL) and the Johns Hopkins University International Consortium for Brain Mapping (JHU ICBM)-DTI-81 white matter labels atlas used in the study.**


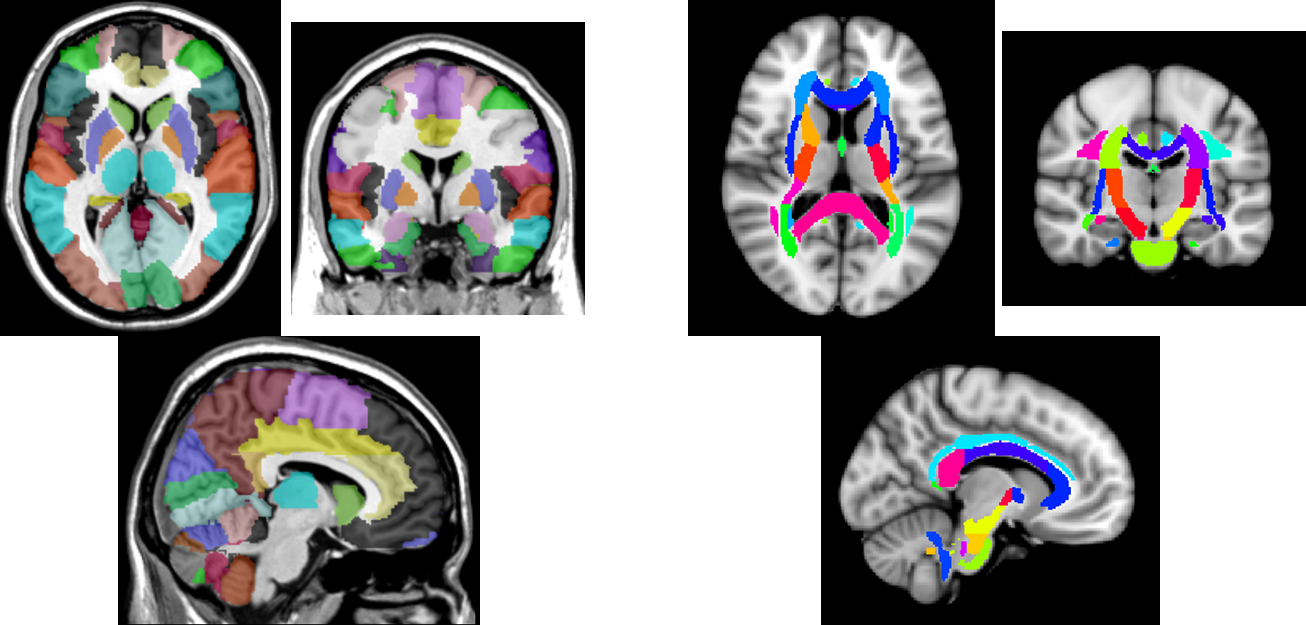

Supplement: Supplementary file 1 — Supplementary material. [file mmc1.doc]
