# Supplementary material for: Magnetic Resonance Imaging Measures of Brain Structure to Predict Antidepressant Treatment Outcome in Major Depressive Disorder
Source: eBioMedicine. 2014 Dec 3;2(1):37–45. doi: 10.1016/j.ebiom.2014.12.002 (PMC4484820; doi:10.1016/j.ebiom.2014.12.002)
Supplement: Supplementary file 2 — Supplementary material. [file mmc2.pdf]

Williams et al. Trials 2011, 12:4
http://www.trialsjournal.com/content/12/1/4

TRIALS

STUDY PROTOCOL Open Access

International Study to Predict Optimized Treatment for Depression (iSPOT-D), a randomized clinical trial: rationale and protocol

Leanne M Williams[1,2*], A John Rush[3], Stephen H Koslow[1,4], Stephen R Wisniewski[5], Nicholas J Cooper[6], Charles B Nemeroff[7], Alan F Schatzberg[8], Evian Gordon[2,6]

Abstract

Background: Clinically useful treatment moderators of Major Depressive Disorder (MDD) have not yet been identified, though some baseline predictors of treatment outcome have been proposed. The aim of iSPOT-D is to identify pretreatment measures that predict or moderate MDD treatment response or remission to escitalopram, sertraline or venlafaxine; and develop a model that incorporates multiple predictors and moderators.

Methods/Design: The International Study to Predict Optimized Treatment - in Depression (iSPOT-D) is a multi-centre, international, randomized, prospective, open-label trial. It is enrolling 2016 MDD outpatients (ages 18-65) from primary or specialty care practices (672 per treatment arm; 672 age-, sex- and education-matched healthy controls). Study-eligible patients are antidepressant medication (ADM) naïve or willing to undergo a one-week wash-out of any non-protocol ADM, and cannot have had an inadequate response to protocol ADM. Baseline assessments include symptoms; distress; daily function; cognitive performance; electroencephalogram and event-related potentials; heart rate and genetic measures. A subset of these baseline assessments are repeated after eight weeks of treatment. Outcomes include the 17-item Hamilton Rating Scale for Depression (primary) and self-reported depressive symptoms, social functioning, quality of life, emotional regulation, and side-effect burden (secondary). Participants may then enter a naturalistic telephone follow-up at weeks 12, 16, 24 and 52. The first half of the sample will be used to identify potential predictors and moderators, and the second half to replicate and confirm.

Discussion: First enrolment was in December 2008, and is ongoing. iSPOT-D evaluates clinical and biological predictors of treatment response in the largest known sample of MDD collected worldwide.

Trial registration: International Study to Predict Optimised Treatment - in Depression (iSPOT-D) **ClinicalTrials.gov Identifier:** NCT00693849

URL: http://clinicaltrials.gov/ct2/show/NCT00693849?term=International+Study+to+Predict+Optimized+Treatment+for+Depression&rank=1

Background

Major depressive disorder (MDD) is the fourth most disabling medical condition worldwide (based on disability-adjusted lifeyears) and is expected to be ranked second by year 2020 [1,2]. MDD is typically recurrent, often chronic and disabling, with a lifetime prevalence rate of over 15% [3]. Women are approximately twice as likely to develop MDD as men. MDD is associated with high health care costs [4]. Antidepressant medications (ADMs) are effective, [5-9], but only about 50% of patients with MDD show a response (>50% reduction in baseline symptoms) and only about one in three attain remission (virtual absence of symptoms) within the first eight weeks of treatment [10-12]. Those who do not attain remission remain at high risk for subsequent depression, functional impairment and serious general

* Correspondence: lea.williams@brainnet.net
[1]BRAINnet Foundation, 71 Stephenson Street, Suite 400, San Francisco, CA, 94105, USA
Full list of author information is available at the end of the article

BioMed Central

© 2011 Williams et al; licensee BioMed Central Ltd. This is an Open Access article distributed under the terms of the Creative Commons Attribution License (http://creativecommons.org/licenses/by/2.0), which permits unrestricted use, distribution, and reproduction in any medium, provided the original work is properly cited.

medical conditions (GMCs) [13-18]. Several treatments for MDD are available, but they are currently selected using a trial-by-trial approach because the field has yet to identify clinically-useful patient baseline measures that reliably recommend one treatment over another (moderators) [19-21]. However, several baseline features that foretell overall outcome regardless of treatment type (predictors) have been identified [22-25].

The ongoing International Study to Predict Optimized Treatment - in Depression (iSPOT-D) is designed to evaluate a range of potentially useful moderators and/or predictors within a group of representative outpatients with nonpsychotic MDD. iSPOT-D is a 'practical trial' [26,27] in that it aims to mirror clinical practice in a representative spectrum of outpatients (to enhance generalizability). In addition to symptoms, iSPOT-D analyzes a range of outcomes including function, adverse events, and side effect burden.

The primary aims of iSPOT-D are to:

1. Identify overall predictors of treatment outcome (response or remission) after up to eight weeks of ADM treatment with escitalopram, sertraline or venlafaxine
2. Identify moderators of treatment outcome (response or remission) after up to eight weeks of ADM treatment
3. Develop a model to incorporate the effects of multiple predictors or moderators on response and remission
4. Conduct a replication study that utilizes the second half of the sample to replicate and confirm the results of the analyses of the first half (Aims 1-3).

Secondary aims include determining predictors and moderators of (1) treatment response according to MDD subtype and (2) symptom severity over time within the primary study period (baseline to week 8) and over the more exploratory follow-up period of 12 to 52 weeks.

In addition, a brain imaging sub-study is assessing 10% of participants and matched controls with magnetic resonance imaging (MRI) and diffusion tensor imaging (DTI) under rest conditions, and functional MRI under task conditions, to evaluate neuroanatomical and neural circuitry measures for diagnostic sensitivity and state versus trait-like effects from baseline to week 8.

Methods/Design

Organizational Structure

The infrastructure of the iSPOT-D multi-centre, international, randomized, prospective, open-label trial includes the Global Coordinating Center and Data Center (Sydney) with Global Trial Coordinator and executive management team; a Molecular Center (Indianapolis); and 20 clinical sites (see Appendix 1), each with a Principal investigator(s) and Clinical Trial Coordinator (CTC). Study clinical sites include clinical research sites within academic settings and clinical sites in clinical practices. Monitoring visits at each clinical site are conducted by Clinical Trial Monitors every eight to 12 weeks (depending on recruitment rates) to ensure procedural and data integrity.

CTCs at each clinical site assist in the recruitment, evaluation, management and assessments of participants. Clinical data are acquired by trained clinicians (psychiatrists or psychologists) who have passed inter-rater reliability training. The iSPOT-D Executive Committee oversees the trial, which is supported by the Clinical Research Organization.

Site selection/training/recruitment

Clinical sites were selected based on the likelihood of meeting recruitment goals and executing the protocol. Most sites are practices that do not typically engage in clinical trials. During a site initiation visit, CTCs at each site are trained and certified in protocol implementation and data collection methods. As new staff is added, they are trained by the Principal Investigator. CTCs work closely with participants and clinicians, administer some clinician-rated instruments, ensure that participants complete all self-rated instruments, and function as study coordinators (i.e., liaise among sites, Clinical Trial Monitors and the Global Coordinating Center).

Study Participants

The iSPOT-D study is ongoing, with enrolment having begun in January 2009. The goal is to recruit 2016 participants with nonpsychotic MDD, with 672 in each of the three treatment groups and 672 age-, sex- and education-matched healthy controls. Broad inclusion and minimal exclusion criteria (Figure 1) are used to recruit representative adult outpatients with nonpsychotic MDD who would typically receive ADM in routine practice. Patients over age 65 are excluded because concomitant medical conditions or medications could interact with protocol medications. Adolescents/children are excluded because the efficacy and safety of most study ADMs have not been established for this age group.

Ethical Considerations

The study is conducted according to the principles of the Declaration of Helsinki 2008 (see Appendix 2) the International Conference on Harmonization (ICH) guidelines (see Appendix 2) and/or in compliance with the laws and regulations of the country in which the research is conducted, including the "Good Clinical Practice" principles in the US FDA Code of Federal Regulations (see Appendix 2).

Inclusion Criteria

- Age 18-65
- Fluent and literate in English or Dutch
- Provide written informed consent
- Total HRSD17 ≤16
- Meets DSM-IV* criteria for single or recurrent nonpsychotic MDD established by MINI Plus

Exclusion Criteria

- Suicidal ideation and/or tendencies, defined by a score ≥8 on Section C of the MINI Plus
- History of bipolar disorder (I, II, not otherwise specified) (lifetime)
- History of schizophrenia, schizoaffective disorder, or psychosis not otherwise specified (lifetime)
- Current primary diagnosis of anorexia or bulimia, obsessive-compulsive disorder, or primary post-traumatic stress disorder
- Known contra-indication for escitalopram, sertraline and/or venlafaxine-XR, or previous treatment failure at the highest recommended dose
- Taking any medication that is contraindicated with escitalopram, sertraline, or venlafaxine-XR
- Taking escitalopram, sertraline, or venlafaxine-XR in the current episode of MDD
- Use of any non-protocol antidepressant drug or CNS drug (antipsychotic, anticonvulsant anxiolytic, clonidine) that cannot be washed out prior to participation
- Has general medical condition that contraindicates protocol antidepressant treatments or interferes with protocol measurements (such as epileptic condition for EEG recording)
- Substance dependence (including alcohol intake equaling 29 standard alcoholic drinks per week for males; >15 for females) in the past six months
- History of brain injury or blow to the head that resulted in loss of consciousness for greater than five minutes
- Severe impediment to vision, hearing and/or hand movement that is likely to interfere with completion of assessments, or with comprehension of instructions or study requirements
- Participation in an investigational study within four months prior to baseline that could affect symptoms of MDD
- Is pregnant or breast-feeding

Figure 1 Inclusion/Exclusion criteria for iSPOT-D study entry. *DSM-IV: Diagnostic and Statistical Manual of Mental Disorders, fourth edition [45]. The term 'primary diagnosis' is used in the context of DSM-IV as shorthand to indicate those mental disorders that are not due to a general medical condition and that are not substance-induced.

Institutional Review Board (IRB) approval is obtained prior to patient enrolment at any clinical site. All protocol modifications are submitted to each IRB for approval before implementation. Prior to undertaking any study-related procedures, each participant receives a verbal and written explanation of study aims, methods, potential hazards and benefits from investigators, and provides written informed consent.

Study Regimens

Enrolment/randomization

Participants are enrolled at each clinical site and randomized to receive escitalopram, sertraline, or venlafaxine-XR as these ADMs are commonly used in practice http://www.guidelines.gov and have distinct pharmacological properties which may enable the identification of moderators. Randomization is carried out using Phase-Forward's™ validated, Web-based Interactive Response Technology. A blocked randomization procedure (block size of 12) is undertaken at the level of the Global Coordinating Center, given that treatment options are equipoise across sites. Open treatment is used to ensure safety and represent clinical practice.

Treatment visits and follow ups

Clinical visits are required at week 0 (baseline) and week 8. Telephone monitoring is undertaken at weeks 2, 4 and 6 to obtain measures of the primary and secondary outcomes. Telephone monitoring with these same

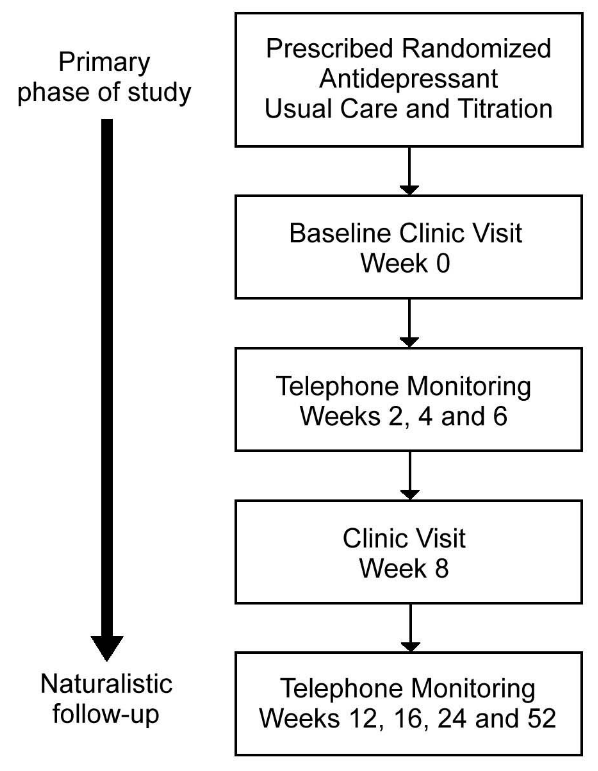

Figure 2 Summary of iSPOT-D monitoring of participants.

measures is continued in the follow-up period at weeks 12, 16, 24 and 52 (Figure 2).

Protocol Treatment Delivery

iSPOT-D aims to ensure representative but high quality treatment implementation and maximal participant retention by collaborating with treating clinicians. Doses for ADM medications are adjusted by the treating clinicians according to routine clinical practice, within the following dose ranges: escitalopram (10 to 20 mg/day), sertraline (50 to 200 mgday) and venlafaxine-XR (75 to 225 mg/day). Participants are compensated for each research assessment (equivalent to $25/1-hour assessment). CTCs remain in contact with participants to enhance participation and minimize premature discontinuation. Newsletters and updates are provided to participants on a monthly basis via e-mail and teleconference, respectively, to maintain interest and motivation and to enhance shared learning.

CTCs at each clinical site perform protocol-specified data gathering and enter data in an electronic case report form (eCRF) after each clinic visit and telephone contact. An investigator site file containing all relevant clinical procedures and study-related documentation has been supplied to each site to provide clear instructions on all relevant clinical procedures, including eCRF completion; psychiatric rating scales; how to perform ECGs; Serious Adverse Event (SAE) reporting; example templates of source document collection forms; and logs used to assist in tracking patient screening, enrolment and discontinuation targets.

Concurrent Treatments

The study allows additional treatments for associated symptoms (e.g., insomnia) or medication side effects (e.g., sexual dysfunction) to reflect common practice. Participants may receive any treatment for concurrent GMCs except medications contraindicated for the use of escitalopram, sertraline or venflaxine-XR. The study proscribes any concurrent medication likely to affect brain function recordings and those that cannot be washed out, including antipsychotics, anticonvulsants, anxiolytics and clonidine. Data on concomitant medication are recorded.

Follow-up

All participants are encouraged to continue the same type and dose of ADM used in the 8 week acute treatment period and to provide telephone-acquired data at weeks 12, 16, 26 and 52.

Data Collection

Self-report questionnaires and tasks used in cognitive and electrical brain and autonomic recordings are each based on well-established constructs in the literature. Unlike previous trials and experimental research in which these constructs were assessed using methods that vary across sites and laboratories, iSPOT-D uses a Web- and computer-enabled infrastructure to acquire data in a standardized way across participants and sites. Self-report data are acquired using standardized Web-based questionnaires, cognitive data are obtained using a standardized computerized touchscreen platform, and electrical brain and autonomic data are acquired using standardized hardware and software (see Appendix 2) [28]. The Brain Resource International Database has been established using this standardized infrastructure [21,29,30], which provides a systematic frame of reference for quality control in the acquisition of all iSPOT-D data.

Screening and Clinical Data

At screening, CTCs gather participant eligibility and sociodemographic data. The Mini-International Neuropsychiatric Interview (MINI-Plus) [31,32] is used to confirm DSM-IV criteria for nonpsychotic MDD, and assess for psychiatric and substance abuse disorders and other potential exclusion criteria. Depressive symptom severity is rated using the 17-Item Hamilton Rating Scale for Depression ($HRSD_{17}$) [33] and the 16-item Quick Inventory of Depressive Symptomatology-Self Report ($QIDS\text{-}SR_{16}$) [34-36] (Table 1).

Clinic Visit Moderator and Predictor Data

Molecular Data At baseline, two 6 mL blood samples are obtained for genotyping. Initial analyses target

Table 1 Data Collection at Baseline Screening

Domain	Measure	Time (minutes)	Method	Administrator
Consent	Consent	15	Interview	CTC
Eligibility	Inclusion/Exclusion	5	Interview	CTC
Psychiatric diagnoses	MINI-Plus	45	Interview	CTC
Symptoms	$HRSD_{17}$	15	Interview	CTC
	$QIDS\text{-}SR_{16}$		Web self-report	Part
Characteristics	Demographics Medical History	10	Web self report	Part.

Abbreviations:

CTC = Clinical Trial Coordinator.

MINI-Plus = Mini International Neuropsychiatric Interview - Plus.

$HRSD_{17}$ = 17-item Hamilton Rating Scale for Depression.

$QIDS\text{-}SR_{16}$ = 16-item Quick Inventory of Depressive Symptomatology - Self-Rated.

Part. = Participant.

300 candidate single nucleotide polymorphisms (SNPs) that might predict or moderate response to antidepressant medication, including 5HT-2A rs7997013 AA allele, 5HT-2A 102T/CC and -1438A/G G alleles, GRIK4 rs1954787gene, tryptophan hydroxylase (TPH) A218C C allele [37-39], FKBP5 [40] and CRF1 [41]. Others have been implicated as a biomarker for non-response to treatment, including 5HTT-LPR short allele [42], HTR1A (rs6295) - 1019 G allele, COMT (val108/158met) Val allele, and the BDNF (brain derived neurotrophic factor) [38,39,42-44]. These candidate genomic variants have also been found to impact the electrical brain, autonomic and brain imaging measures used in iSPOT-D in relation to depression [45-47]. Sufficient blood is collected to also explore gene expression, proteomics and metabolomics. Urine samples are obtained to provide data on illicit drug use and rule out other prescription medications (Table 2).

Clinical, Functional Status, and Disposition At the baseline and week 8 clinic visits, participants complete the self-report $HRSD_{17}$ (primary clinical outcome measure) (see Appendix 2) and secondary outcome measures including the $QIDS\text{-}SR_{16}$ to assess depressive symptom severity, the Frequency and Intensity Burden of Side Effects Rating (FIBSER) [48], the World Health Organization Quality Of Life (WHOQoL) scale [49], the Social Functioning and Adjustment Scale (SOFAS) [50], and the Emotion Regulation Questionnaire (ERQ) [51] to assess functional status and measures of disposition (Table 2). These secondary outcome measures are included in the Web-based questionnaire battery (see Appendix 2). The $QIDS\text{-}SR_{16}$ and the FIBSER are also collected during the telephone monitoring sessions in the acute and follow-up phases (Figure 2) (see Appendix 2).

Measurement of Self Regulation and Feeling Processes The Web-based questionnaire battery (baseline and week 8) includes the Brain Resource Inventory of Social Cognitions (BRISC) [28,30,47] for assessment of self-regulation processes. The BRISC contains 45 self-report items from which scores are obtained for: ***Negativity Bias***, the tendency to see oneself and one's world as negative; ***Emotional Resilience***, self-confidence and the capacity for coping with life; and ***Social Skills***, the capacity for building and maintaining relationships (Table 3). The BRISC has been normed and validated with regard to its biological basis [28,30,47]. Participants also complete the full version of the Depression Anxiety and Stress Scales (DASS), a 42-item instrument that yields measures of depression, anxiety and stress [52,53], which has been normed internationally [54].

Cognitive Data for Emotion and Thinking Processes At baseline and week 8, participants complete cognitive tasks that assess Emotion and Thinking processes. Within the Emotion domain, there are two sub-domains: emotion identification and emotion recognition (Table 4). The tasks assessing these sub-domains are, respectively: explicit emotion identification and implicit emotion recognition [55], yielding both accuracy (error rates) and reaction time measures. Participants complete these cognitive tasks using a standard, computerized touchscreen platform (see Appendix 2) [21,29,30] which does not rely on keyboard or computer skills. Standardized task instructions are concurrently presented visually on the screen and via headphones. Reaction time and accuracy are recorded via the touchscreen computer and verbal responses via a microphone and recording system attached to the headphones. Psychometric properties have been established, including large norms, validation against traditional paper and pencil tests tapping equivalent domains, test-retest reliability, and consistency across cultures [55-60]. Biological validation against brain measures has also been established in the same participants [56,59,61-63]. The touchscreen cognitive assessments have demonstrated utility in clinical groups [64-69].

Within the Thinking domain, there are six sub-domains each assessed by at least one task: response speed, impulsivity, attention-concentration, information

Table 2 Data Collection at Clinic Visits (Baseline and Week 8): Symptom, Functional Status, Disposition and Molecular data

Domain	Measure	Time (minutes)	Method	Administrator
Symptoms	$QIDS\text{-}SR_{16}$	5	Web Self report	Part.
Side Effects	FIBSER	1	Web Self report	Part.
Functional Status	WHOQOL	5	Web Self report	Part.
	SOFAS	3	Web Self report	CTC
	SWLS	1	Web Self report	Part.
	ERQ	5	Web Self report	Part.
Disposition	Early Life Stress	3	Web Self report	Part.
	Personality traits (NEO-FFI)	5	Web Self report	Part.
Molecular*	Genomics	10	Blood draw	CTC
	Drug Screen	8	Urine sample	Part.

Abbreviations:
$QIDS\text{-}SR_{16}$ = 16-item Quick Inventory of Depressive Symptoms - Self-Report.
Part. = Participant.
FIBSER = Frequency, Intensity and Burden of Side Effects Rating.
WHOQOL = World Health Organization Quality of Life.
SOFAS = Social Functioning and Adjustment Scale.
SWLS = Satisfaction With Life Scale.
ERQ = Emotion Regulation Questionnaire.
CTC = Clinical Trial Coordinator.
NEO-FFI = NEO Five Factor Inventory.

processing efficiency, memory, and executive functioning (Table 5). The tasks that assess each sub-domain yield error, reaction time and task-completion-time measures.

Electrical Brain and Autonomic data At baseline and week 8, electrophysiological measures are acquired using standard pre-specified hardware and software to acquire data on electroencephalogram (EEG), event-related potentials (ERPs) elicited by activation tasks, and concurrent autonomic measures of heart rate function and eye blinks (Table 6).

Electrical brain data Resting EEG and task-activated ERP data are recorded continuously from 26 scalp sites with a NuAmps system and QuickCap. Horizontal and vertical eye movement electrodes are placed near the eyes.

Resting EEG The resting EEG is recorded for two minutes while participants are relaxed with eyes open. Alpha asymmetry, which has been implicated in depression [70], is computed by subtracting Alpha power for a left scalp sites (e.g., left fronto-central sites F3, FC3) from the homologous right sites (F4, FC4), and dividing this difference by their sum.

Positive values reflect greater right versus left frontal alpha power, indicating relatively greater *left* frontal activity, since higher alpha power has traditionally been interpreted as reflecting less cortical activation [70,71]. Maximal asymmetry is indicated with 1.0 and maximal symmetry is indicated with 0.

Activation task-elicited ERPs ERP components are elicited by each activation task and defined by published criteria [28,58,72] (Table 7). The primary ERP for each

Table 3 Data Collection at Clinic visits (Baseline and Week 8): Self-Regulation and Feeling data

Domain	Measure	Time (minutes)	Method	Administrator
Self Regulation	Negativity Bias[a]	5	Web Self report	Part.
	Emotional Resilience[b]		Web Self report	Part.
	Social Skills[b]		Web Self report	Part.
Feeling	DASS Depression[a]	7	Web Self report	Part.
	DASS Anxiety[a]		Web Self report	Part.
	DASS Stress[a]		Web Self report	Part.

[a] Hypothesized contrast is Treatment Responders > Non-Responders.
[b] Hypothesized contrast is Treatment Responders < Non-Responders.
Abbreviations:
Part. = Participant.
DASS = Depression Anxiety and Stress Scale.

Table 4 Data collection at Clinic visits (Baseline and Week 8): Cognitive data for Emotion processes

Domain	Sub-domain	Task	Measure[a]	Time (minutes)	Method	Administrator
Emotion	Emotion Identification	Explicit Emotion Identification[b]	Fear Errors	8	Touchscreen	Part.
			Fear Reaction Time			
			Anger Errors			
			Anger Reaction Time			
			Sad Errors			
			Sad Reaction Time			
			Disgust Errors			
			Disgust Reaction Time			
			Happy Errors			
			Happy Reaction Time			
			Neutral Errors			
			Neutral Reaction Time			
Emotion	Emotion Recognition	Implicit Emotion Recognition[c]	Fear Errors		Touchscreen	Part.
			Fear Reaction Time			
			Anger Errors			
			Anger Reaction Time			
			Sad Errors			
			Sad Reaction Time			
			Disgust Errors			
			Disgust Reaction Time			
			Happy Errors			
			Happy Reaction Time			
			Neutral Errors			
			Neutral Reaction Time			

[a] Hypothesized contrast is Treatment Responders < Non-Responders.
(with a common direction of reduction for poorer accuracy and slowed reaction time).
[b] Participant identifies the verbal label for each facial emotion.
[c] Facial emotions are represented and participants determine whether or not they have seen each face before.
Abbreviations:
Part. = Participant.

task is quantified as the maximum amplitude (in microvolts) of the change in potential from pre-stimulus baseline, averaged across task trials to obtain a single value for each participant.

Autonomic data

Resting heart rate Heart rate is based on an electrocardiogram (ECG) (sampling rate of 500 Hz) with electrodes positioned on the inner left wrist at the radial pulse and on the right clavicle. ECG is recorded concurrently with the EEG during the entire resting condition.

Activation Task Heart Rate Heart rate is obtained concurrently with ERPs during the Oddball, Continuous Performance, Novelty, Go-No Go and Masked and Unmasked Emotion tasks (Table 6). Mean heart rate is quantified as beats per minute for the duration of each task, which allows a calculation of mean heart rate change and heart rate variability change between resting and task conditions.

Brain imaging data Ten percent of participants provide brain imaging data including structural MRI, functional MRI and DTI, using 3Tesla scanners. Functional MRI is undertaken with the same Oddball, Go-No Go, Continuous Performance and Emotion tasks used for ERP recording. Brain imaging recording will be completed at Baseline and Week 8. A more comprehensive description of the brain imaging data will be presented in a subsequent report.

Research Outcomes

The primary research outcome is treatment response, defined as a ≥50% decrease from the baseline $HRSD_{17}$. Secondary outcomes include remission, defined as a score of ≤7 on the $HRSD_{17}$. The secondary endpoint for remission is a score of ≤5 on the $QIDS\text{-}SR_{16}$. Additional secondary outcomes include depressive symptoms ($QIDS\text{-}SR_{16}$), side-effect burden (FIBSER), WHO quality of life (WHOQoL), social functioning and adjustment scale (SOFAS), satisfaction with life scale (SWLS) and the emotion regulation questionnaire (ERQ).

Data Management

Data upload and transfer Data from the eCRF are entered by site staff into each site's InForm database, which are coordinated using the PhaseForward InForm

Table 5 Data collection at Clinic visits (Baseline and Week 8): Cognitive data for 'Thinking' processes

Domain	Sub-domain	Task	Measure[a]	Time (minutes)	Method	Administrator
Thinking	Response Speed	Motor Tapping	Number of Taps	30	Touchscreen	Part.
			Variability of Pause between Taps			
	Impulsivity	Go-NoGo	Reaction Time	30	Touchscreen	Part.
			Variability of Reaction Time			
			False 'alarm' errors			
	Attention-Concentration	Continuous Performance Test	Reaction Time	30	Touchscreen	Part,
			False 'alarm' errors			
			False 'miss' errors			
	Information Processing Efficiency	Switching of Attention	Completion Time (digits + letters)	30	Touchscreen	Part.
			Average Connection Time (digits + letters)			
			Errors (digits+letters)			
		Verbal Interference	Part 2- Part 1 Errors	30	Touchscreen	Part.
			Part 2- Part 1 Reaction Time			
		Choice Reaction Time	Reaction Time	30	Touchscreen	Part.
	Memory	Digit Span	Recall Span	30	Touchscreen	Part.
			Trials correct			
		Memory Recognition	Total immediate recall trials 1-4	30	Touchscreen	Part.
			Learning rate trials 1-4			
			Delayed recall trial 7			
	Executive Function	Maze	Completion Time	30	Touchscreen	Part.
			Path Learning Time			
			Overrun Errors			
			Total Errors			

[a] Hypothesized contrast is Treatment Responders < Non-Responders.
(with a common direction of reduction for poorer accuracy, reduced variability, and slowed reaction time).
Abbreviations:
Part. = Participant.

protocols and are accessible by the Global Coordinating Center with password control. The source documents are retained by each site and will be archived for 15 years beyond study completion, or in accordance with local regulations, whichever is longest.

Blood samples collected for genomics at each site are placed immediately into a freezer at -20 degrees Celsius or colder. They are then sent on dry ice to the Covance Molecular Coordinating Center (MCC) at Indianapolis, where they are stored at -70 degrees Celsius. Samples from non-US sites are forwarded to Indianapolis via initial storage at Covance sites in Geneva or Singapore.

For the Web-based questionnaire, each self-reported response entered by participants is logged. For the touchscreen-based cognitive tests, the computer registers each touch, press and drag made by the user as each task is performed and writes these with time stamps to a log file. Electrical brain and autonomic data are recorded onto the computer as participants complete each condition and task. The computer registers each datapoint every 2 ms and writes these data with a time-stamped log file. All these data are part of the standard computerized Brain Resource data acquisition infrastructure, which connects to the Web-enabled data upload system. Once the CTC clicks 'submit', the data are instantly uploaded as an xml file to the Upload Server. From there, data are transferred to the 'Scoring Server' (see Data Quantification sub-section) and then into the Data Center database at the Global Coordinating Center (see Data Storage sub-section).

Data Storage The InForm databases from each site are collated into the central Data Center database. Quantified data are written to a robust relational DB2 database, designed to accept the quantified data from all data modalities. Following quantification, all other measures are written to the DB2 database. In each modality, quantified data for storage is in the form of a numerical value. The database is designed to be scalable and expandable throughout its life. After completion of the final participant visit, the Global Trial Coordinator will lock the data.

Data quantification A dedicated 'Scoring Server' is in place for quantifying each type of data for iSPOT-D. The

Table 6 Data collection at Clinic visits (Baseline and Week 8): Electrophysiological and Autonomic data

Domain	Task	Measure*	Time (minutes)	Method	Administrator
**Electrophysiological**					
Resting condition	Eyes Open	Frontal Alpha Asymmetry[a]<br>Fronto-parietal Alpha power[a]<br>Fronto-parietal Theta power[b]	2	EEG	CTC
Activation tasks	Oddball	Fronto-Parietal P300 ERP[a]	6	ERP	CTC
	Continuous Performance Test (CPT)	Frontal P450 ERP[a]	8	ERP	CTC
	Novelty	Frontal Early P300 ERP[a]			
	Go-No Go	Frontal N200 ERP[a]	6	ERP	CTC
	Emotion (masked, nonconscious)	Temporo-occipital P120, Fronto-central VPP ERP[b]	6	ERP	CTC
	Emotion (unmasked, conscious)	Temporo-occipital P120, Fronto-central VPP ERP[b]	6	ERP	CTC
	Startle 'noise burst'	Fronto-central N100-P200 ERP[b]	4	ERP	CTC
**Autonomic**					
Resting condition	Eyes Open	Average Heart Rate[a]<br>Heart Rate Variability[a]		ECG	
Activation tasks	Startle	Eye blink[b]		EMG	CTC
	Oddball, CPT, Novelty, Go-NoGo, Emotion (masked, unmasked)	Average Heart Rate, Heart Rate Variability[a]<br>Average Skin conductance level[a]		ECG Electro-dermal activity	

[a] Hypothesized contrast is Treatment Responders < Non-Responders.
[b] Hypothesized contrast is Treatment Responders > Non-Responders.
Abbreviations:
Part. = Participant.
CTC = Clinical Trial Coordinator.
CPT = Continuous Performance Test.
EEG = Electroencephalogram.
ERP = Event Related Potential.
EMG = Electromyogram (Orbicularis Occuli).
ECG = Electrocardiogram.
*These are representative measures based on previous findings; additional EEG measures from other recording sites may also be analyzed.

'Scoring Server' implements criteria for screening of data quality and quality control (see Section 6.5 for quality control details).

Clinical data uploaded to the Data Center from the InForm database are quantified according to the scoring manuals for each assessment. Genotyping is undertaken using a standardized array, which allows custom genotyping of SNPs within candidate genes or genomic intervals. Genotyping is expressed as number of alleles (allele loading), coded according to number of a particular allele, as 0, 1 or 2 (corresponding to 2, 1 or 0 of another allele respectively).

Web-based questionnaire data is quantified automatically by the Scoring Server, which has been programmed with the manual scoring criteria for each scale. Cognitive data is quantified into reaction time and error scores automatically using a software program. Verbal responses recorded via sound files are automatically collated by the server and allocated to pre-certified trained scorers, who use text fields (as per dictionary included 'real' words) to transcribe participant responses into the server. Transcription is verified by an independent scorer.

The Scoring Server for Electrical Brain and autonomic data (in the form of 'Neuroscan 5' files) includes a series of artifact correction and rejection procedures. Low and high frequency noise is removed by high-pass and low-pass filters, power line artifact by notch filters, and muscle and blink artifact by second-order blind identification and canonical correlation analysis. The Scoring Server also includes quality control software that detects five additional primary sources of artifact (using thresholds for abnormal voltage, baseline shifts and kurtosis) for removal prior to quantifying the data.

EEG, ERP and autonomic measures are quantified by algorithms in the Scoring Server Software that have been verified against the gold standard of manual scoring with high inter-scorer reliability. Consensus criteria for quantification are used [73].

Table 7 Activation Task-Elicited Event Related Potentials

Event Related Potential[a]	Description	Participant Response	Measure definition	Analysis
Oddball P300[b]	Series of 300 tones presented at 75 db (each 50 ms, ISI = 1 second)	Press a button in response to high-pitched tones (1000 Hz), ignore low-pitched tones (500 Hz)	P300 over the parietal cortex ≈300 ms after each target stimulus (range: 270-450 ms)	Amplitude averaged across target trials for frontal and parietal recording sites, Fz and Pz
Continuous Performance P450[b]	Series of 125 letters (B, C, D or G) presented sequentially (each 200 ms, ISI = 2.5 seconds)	Press a button when the same letter appears twice in a row	P450, occurring in response to updating of non-target letters at ≈450 ms after letter (range: 300-550 ms). Most prominent over frontal brain regions (71)	Averaged across non-target letter trials for the frontal Fz recording site
Novelty Early P300	Series of 20 blue and green checkerboard stimuli presented briefly (200 ms) and infrequently, unexpectedly and at random intervals within the Continuous Performance Test (ISI = 2 seconds)	No response required	P300 occurring 250 ms after novelty stimuli over medial-central frontal brain regions (range: 220-320 ms) (72)	Averaged over novelty trials and over Fz and Cz recording sites
NoGo N200[b]	168 stimuli presented sequentially (200 ms each, ISI = 2 seconds)	Press a button as quickly as possible for Go stimuli, don't press for NoGo stimuli	N200 occurring ≈200 ms after NoGo stimuli over fronto-central brain regions (range: 150-230 ms) (39)	Elicited within the timescale of 'automatic' error detection and impulsivity (73)
Emotion P120 and Emotion VPP	Series of 288 stimuli presented (3-dimensional facial expressions depicting fear, anger, disgust, sadness, happiness or neutral) (500 ms each, ISI = 767 ms)	Active viewing, no response required.	P120 occurring around 120 ms over temporo-occipital sites (range: 80-140 ms)<br>VPP occurring around 170 ms over fronto-central sites (range: 120-220 ms)	Average of 32 estimuli for each emotion for P120 (T5, T6, O1, O2 sites); VPP Fz, Cz sites)
Startle "noise burst"	Series of 20 acoustic startle stimuli (white noise burst at 105 db, 50 ms duration, ISI = 10-15 seconds)	Startle eye blink: muscle contraction of the eye blink reflex as measured by the electromyogram (82)	Onset latency, peak amplitude and peak latency	Averaged across the 20 trials, excluding non-response trials

[a] Name of ERP component indicates polarity direction in which the change in potential occurs (P = positive, N = negative, and the number represents the approximate time [in ms] at which it occurs after each stimulus.

[b] Participant informed that speed and accuracy are important to the task.

Abbreviations:

ISI = Interstimulus Interval.

Quality Control

All data is de-identified using an 8-digit identification number - with a session-number suffix - to provide privacy and confidentiality in accord with relevant guidelines (see Appendix 2). iSPOT-D has a Quality Control Review Record to record any queries about the data and any changes, with reasons. This record will be kept for at least two years after datalock [74].

Training Quality control of training is overseen by the Global Coordinating Center. Each CTC is trained on-site in data acquisition using the eCRF; the MINI-Plus and $HRSD_{17}$ with paper forms; and the standardized protocols for the Web-based questionnaire, touchscreen cognitive assessments, and electrophysiological and autonomic recordings. For accreditation, each CTC must perform at least one acquisition under the supervision of the Global Trial Coordinator (or delegated staff from the executive management) and provide three complete datasets that meet the quality control criteria of the Data Center.

Acquisition and Upload Quality control for clinical data acquisition is overseen by a Clinical Research Organization, PhaseForward, which has on-line data checks which are activated as soon as the data is submitted to the InForm database. A Clinical Trial Monitor performs on-site Source Document Verification to confirm accurate data entry for samples of data, in accordance with the Monitoring Plan.

Procedural quality control of clinical data is undertaken in accordance with ICH Good Clinical Practice (ICH-GCP) guidelines, overseen by the Global Trial Coordinator. These controls include safeguarding the blinding, maintaining a secure system that prevents unauthorized access to the data, and managing a secure and audited system that permits authorized changes which are documented and ensure that no entered data is deleted. Within these guidelines, an audit trail tracks each data entry to the InForm database and precludes changes to the data once entry is confirmed. Reports are

provided to the Data Center confirming the quality control on each participant in iSPOT-D.

Inter-rater reliability for the primary outcome measure ($HRSD_{17}$) is audited for each clinician at each testing site annually, using an established video-based methodology [75]. Clinicians who differ from the average across sites are advised by the head statistician at the Global Coordinating Center of how their rating on applicable items differs to others and they are allowed to re-sit the rating exam until they are able to rate within the bounds of the combined site group. The quality control of blood sample storage is monitored by the well-established protocols of Covance laboratories at the MCC in Indianapolis.

Quality control for the acquisition and upload of Web-based questionnaire, touchscreen, electrophysiological and autonomic data is incorporated in the Upload Server and Scoring Server software. These protocols also meet the specifications of the ICH-GCP. Recording channels with confirmed artifact are set as missing in the study database. The senior technician has the authority to override an automatic score in the case of discrepancy.

For quality control of data scoring, at least 10% of data for each measure is de-identified, reprocessed and compared to the original results to ensure reproducibility with zero-tolerance for error. For auto-scored EEG and ERP data, trained and accredited hand scorers confirm the quantification against criteria from the established literature (Brain Resource ERP Scoring Manual, 2010). For accreditation, scorers must have ≥50 hours experience and have passed reviews of their scoring by the scoring manager, reporting to the GTC. Any queries or changes must be approved by the Data Center manager and are recorded in the Quality Control Review Record.

Analytic Approach

iSPOT-D has been registered and is being conducted as a single study. However, study aims will be addressed by a two-step analysis procedure.

Aims 1 through 3 will utilize the first half of the sample (n = 1,008) to identify potential predictors and moderators. Aim 4 will utilize the second half of the sample (n = 1,008) to replicate and confirm the results generated from the analyses of the first half. The four aims of the study and hypotheses are as follows:

1. Identify overall predictors of treatment outcome (response or remission) after up to eight weeks of ADM treatment

The predictive effect of baseline characteristics will be assessed overall, controlling for any treatment effect. Regression models will be used to assess the predictive effect of each characteristic on outcome. Independent variables in the model will include main fixed effects for treatment and the possible predictive variable. All effects will be centered to aid interpretability (+1/2 and -1/2 for treatment choice and any binary predictor, deviation from the mean baseline for any ordinal predictor). A baseline characteristic will be considered a predictor if the p-value is <.05.

2. Identify moderators of treatment outcome (response or remission) after eight weeks of ADM treatment

The moderating effect of baseline characteristics will be assessed separately for each pairwise comparison of treatment (escitalopram vs. sertraline, escitalopram vs. venlafaxine-XR, sertraline vs. venlafaxine-XR). Regression models will be used to assess the moderating effect of each characteristic on outcome. Independent variables in the model will include main fixed effects for treatment, the possible moderator variable and the two-way interaction between the characteristic and treatment. All effects will be centered to aid interpretability (+1/2 and -1/2 for treatment choice and any binary moderator, deviation from the mean baseline for any ordinal moderator). A baseline characteristic will be considered a moderator if the p-value is <.05.

3. Develop a model to incorporate multiple predictor or moderator effects on response and remission

Recursive partitioning methods will be used to identify how various baseline characteristics interact with treatment and with each other in their association with treatment response. The recursive partitioning approach will be used to develop a decision tree which selects treatment and baseline characteristics with maximization of the sensitivity and specificity of the decision tree in the prediction of treatment response, while minimizing the complexity of the decision tree. The overall sensitivity and specificity of the tree will be reported, along with 95% confidence intervals for each estimate.

4. Replicate the findings in Aims 1-3

A confirmatory analysis will be conducted and the same models that were fit in Aims 1 and 2 will be fit to the data from the relevant comparison in the replication sample. For example, if gender is identified as a predictor of outcome, then the effect of gender will be examined as a potential predictor in the replication sample. For both the initial model and the replication model, a confidence interval will be estimated for the parameter estimate for the main effect of the potential predictor variable model (Aim 1) or for the interaction term in the model (Aim 2). If the two confidence intervals overlap, the results will be considered partially confirmed.

A decision tree will have been generated using data from the first half of the sample, along with an estimate of the tree's overall sensitivity and specificity, and a calculation of a 95% confidence interval for the sensitivity and specificity. The data from the replication sample

will be applied to the decision tree from the initial sample and the sensitivity and specificity will be calculated along with 95% confidence intervals. If the confidence intervals from the initial and replication samples overlap, the results will be considered partially confirmed.

Specific working hypotheses to test each of the primary aims of the study are listed below. These hypotheses draw on a theoretical integration of the published research evidence. From this evidence, 'candidate markers' for predicting and moderating response to antidepressants have been identified. To date, studies have typically examined one candidate marker of antidepressant response and major depressive disorder at a time, using laboratory-specific measures. By using standardized assessments to assess multiple candidate markers in the same study and same patients, iSPOT-D provides enhanced statistical power to identify which markers contribute the most effect size to predicting and moderating antidepressant response.

For each hypothesis, the primary outcome measure of response to antidepressants is change on the $HRSD_{17}$, and the secondary outcomes are change on the self-reported $QIDS\text{-}SR_{16}$ and functional outcome measures (WHOQoL, SOFAS, SWLS, ERQ):

1. Baseline severity of clinical symptoms will predict acute response to antidepressants, and moderate response to type of antidepressant, at 8-week follow up.
2. Baseline psychological features, including exposure to early life trauma and stress-related temperament, will predict acute response to antidepressants, and moderate response to type of antidepressant, at 8-week follow up.
3. Baseline level of cognitive function on emotion tasks will predict acute response to antidepressants, and moderate response to type of antidepressant, at 8-week follow up.
4. Baseline level of cognitive function on thinking tasks will predict acute response to antidepressants, and moderate response to type of antidepressant, at 8-week follow up.
5. Baseline degree of asymmetry on the EEG measure of Alpha power will predict acute response to antidepressants, and moderate response to type of antidepressant, at 8-week follow up.
6. Baseline degree of heart rate variability on autonomic measures will predict acute response to antidepressants, and moderate response to type of antidepressant, at 8-week follow up.
7. For genetics, the presence of specific SNP alleles will predict acute response to antidepressants, and moderate response to type of antidepressant, at 8-week follow up; the 5HT-2A rs7997013 AA allele, 5HT-2A 102T/CC and -1438A/G G alleles, GRIK4 rs1954787gene, tryptophan hydroxylase (TPH) A218C C allele, FKBP5 and CRF1 will moderate a positive response, and the 5HTT-LPR short allele, HTR1A (rs6295) - 1019 G allele, COMT (val108/158met) Val allele, and the BDNF (brain derived neurotrophic factor) will moderate a non-response.

Analyses are planned to test each of the core aims and hypotheses. The primary outcome measure of response to antidepressants is change on the $HRSD_{17}$. The secondary outcomes are change on the self-reported $QIDS\text{-}SR_{16}$ and functional outcome measures (WHOQoL, SOFAS, SWLS, ERQ). Independent measures of clinical severity, psychological function, EEG and genetics being tested as predictors/moderators of the independent variables are listed in Tables 2 to 7. For genetic predictors and moderators, we have focused on an allele-wise approach to target those SNPs that have reported associations in the literature. Sufficient blood is being collected to also explore genome-wide associations between antidepressant response and predictor/moderator variables in future, unplanned analyses.

Sample Size, Power and Effect Size

The primary goal of the proposed study is to identify a number of characteristics which are differentially associated with outcomes across various treatments. This extends the traditional randomized clinical trials which directly compare treatments or a study designed to specifically test the moderating effect of one or more baseline characteristics. The sample size has been selected to provide statistical power of at least 89% power to detect small effects for predictors (odds ratio 1.3 per standard deviation change in the independent baseline measure) at an alpha level of $p < .05$; 94% power to detect medium effects for predictors (odds ratio of 1.5) at an alpha level of $p < .001$, 94% power to detect medium effects for moderator interaction terms (odds ratio of 1.5) at an alpha level of $p < .01$. In addition to replication in the second 1000 participants, we aim to control type I error by applying effect size criteria for each logistic model that odds ratios for the main parameter of interest must exceed 1.3.

Data Monitoring and Safety Reporting

A data and safety monitoring board (DSMB) meets every two months to monitor various aspects of the study including participant recruitment, protocol compliance, and SAEs. The DSMB comprises a minimum of three members with representation from psychiatrists, primary care physicians and a statistician. Statisticians at the

Global Coordinating Centre monitor the age, sex and education distributions of each group every month to ensure matching (±3 years for age, ±1 year for education).

Site recruitment and retention is monitored weekly by the Global Trial Coordinator and CTC for each site. Monitoring is undertaken in accordance with ICH-GCP guidelines. The Monitoring Plan requires 100% source data verification of SAEs and primary outcome measures, and a sample of cognition and brain data. All informed consents are reviewed. Sites that enrol more participants are monitored more frequently.

The Clinical Research Organization is responsible for ensuring that the rights and welfare of participants are maintained, data quality is satisfactory and the trial is conducted in accordance with ICH-GCP and country-specific guidelines, as well as with the protocol's standard operating procedures.

All SAEs (Figure 3) are recorded in the eCRF and on the "Serious Adverse Event Report" form. All SAE entries indicate whether the SAE is serious, the severity, date of onset, whether it is related to study medication or procedure, the action being taken, and resolution. The Global Trial Coordinator may request additional information from the investigator to ensure the timely completion of accurate safety reports. SAE follow-up continues through the last day on study (including the off-study follow-up medication period) and/or until the Global Trial Coordinator and Principal Investigator for the site determine that the participant's condition is stable. Brain Resource may request that certain SAEs be followed until resolution.

The Principal Investigator ensures that all measures necessary for resolution of the SAE are taken. All medications necessary for treatment of the SAE are recorded in the concomitant medication section of the eCRF.

The investigator notifies the Institutional Review Board or Independent Ethics Committee (in writing) of SAEs as soon as is practical where this is required by local regulatory authorities and in accordance with the local institutional policy. In accordance with the European Union Clinical Trials Directive (2001/20/EC), the Sponsor or its designee notifies the Ethics Committees of the concerned Member States of SAEs that are unexpected and possibly attributable to the treatment medication. In all countries, SAEs are reported in accordance with the regulations governing expedited reporting for registered products. SAE reporting is monitored by the Clinical Research Organization and all SAEs are reviewed by the DSMB.

Serious Adverse Event

Any adverse drug experience occurring at any dose that results in any of the following outcomes:

- Death
- Life-threatening situation (participant is at immediate risk of death)
- Inpatient hospitalization or prolongation of existing hospitalization (excluding those for study therapy or placement of an indwelling catheter, unless associated with other SAEs)
- Persistent or significant disability/incapacity
- Congenital anomaly/birth defect in the offspring of a participant who received treatment medication
- Any medically significant events that may not be immediately life-threatening or result in death or hospitalization, but based upon appropriate medical and scientific judgment may jeopardize the participant or may require medical or surgical intervention to prevent one of the outcomes listed above

Figure 3 Definition of Serious Adverse Events.

Discussion

iSPOT-D, a randomized controlled study, aims to identify moderators and predictors of treatment response among three treatments: escitalopram, sertraline, and venlafaxine-XR. Potential moderators or predictors include measures of depressive symptoms, functional status, side-effect burden, genomic, cognition, brain function and brain imaging. Participants are being recruited from clinical and academic sites to assemble a broadly inclusive and representative population. Thus, study results should be widely generalizable.

iSPOT-D includes an innovation in study design with the use of cognitive, brain and gene measures for the identification of objective markers that may moderate or predict response to ADMs. Identifying these markers will be an important first step in a 'personalized medicine' approach to the management of MDD.

Appendices

Appendix 1

Summary of proposed sites for iSPOT-D

USA

Academic Sites:

Stanford University, Department of Psychiatry
Massachusetts Institute of Technology
Harvard University, McLean Hospital
University of St Louis Missouri, Department of Psychology
Ohio State University
University of Virginia, Center for Psychiatric Clinical Research

Clinical Sites:

Shanti Clinical Trials, Colton, California
Center for Healing the Human Spirit Tarzana, California
Skyland Behavioral Health Associates, North Carolina
NeuroDevelopment Center, Providence RI, Academic affiliation: Brown University
Brain Resource Center, NYC, Academic affiliation: Columbia University

UK
Academic Sites:

Kings College Institute of Psychiatry, London

Netherlands
Clinical Sites:

Brainclinics Diagnostics & Treatment, Nijmegen, Academic affiliation: Nijmegen University

Australia
Academic Sites:

University of Sydney, Westmead Hospital
Monash University, Melbourne, Alfred Hospital
Swinburne University, Melbourne, Brain Sciences Institute
Flinders University, Adelaide, Cognitive Neuroscience Unit

Clinical Sites:

Mind Medico, Tasmania, Academic affiliation: University of Tasmania

New Zealand
Academic Sites:

Auckand University, Department of Psychiatry

Clinical Sites:

Brain Health, Johannesburg, Academic affiliation: University of Wittswatersrand

Appendix 2

Regulations/Guidelines, Trademark Names, and Outcome Measure Details

Regulations/Guidelines

World Medical Association Declaration of Helskini: http://www.wma.net/en/30publications/10policies/b3/index.html

ICH Guidelines: http://www.ich.org/home.html

FDA Code of Federal Regulations:

http://www.fda.gov/ScienceResearch/SpecialTopics/RunningClinicalTrials/ucm114928.htm

http://www.accessdata.fda.gov/scripts/cdrh/cfdocs/cfcfr/cfrsearch.cfm?cfrpart = 312

European Medical Association: http://www.ema.europa.eu/pdfs/human/ich/013595en.pdf

Australia; Therapeutic Goods Association regulations:
http://www.tga.gov.au/docs/html/ich13595.htm

New Zealand; Medsafe Good Clinical Practice Guideline and Codes:
http://www.medsafe.govt.nz/profs/regissues.asp

South Africa; Department of Health guidelines:
http://www.doh.gov.za/docs/policy/trials/trials_01.html

Trademark Names for Brain Resource Data Acquisition Methods

- Web-based battery of self-report questionnaires: WebQ™
- Brain Resource Inventory of Social Cognitions: BRISC™. The BRISC is a Web-based battery implemented in conjunction with WebQ.
- Computerized cognitive test battery operating on a touchscreen platform: IntegNeuro™. The version of 'IntegNeuro' that operates in conjunction with LabNeuro (listed below) has also been called 'Psychometrics'.
- Computerized resting and task conditions for recording of EEG, ERPs and autonomic data: LabNeuro™.
- Standardized sequences and software for MRI, functional MRI and DTI: MRI-Neuro™.
- Standardized protocols for acquiring and transporting DNA samples for genotyping; MolecularNeuro™.

Outcome Measure Details

The supplementary four items contributing to the 21-item version of the $HRSD_{17}$ will also be assessed, but not used as part of the primary outcome score.

Reasons for exit before 8 weeks are recorded. $QIDS\text{-}SR_{16}$ data will be available for these participants, for the weeks prior to week 8.

Acknowledgements
iSPOT-D is sponsored by Brain Resource Company Operations Pty Ltd. Brain Resource has established a Publication Committee, chaired by AJR, to provide transparent oversight and management of publication of results from the study, with the participation of study site investigators.
We acknowledge the iSPOT-D Investigators Group, and the contributions of principal investigators at each site. We gratefully acknowledge the editorial support of Jon Kilner, MS, MA (Pittsburgh, PA, USA), the Scoring Server management by Donna Palmer, PhD (Brain Resource) and the monitoring support of PhaseForward.

Author details
[1]BRAINnet Foundation, 71 Stephenson Street, Suite 400, San Francisco, CA, 94105, USA. [2]Westmead Millennium Institute, University of Sydney Medical School, Westmead Hospital, NSW 2145, Australia. [3]Duke-NUS Graduate Medical School Singapore, 8 College Road, 169857, Singapore. [4]American Foundation for Suicide Prevention, 120 Wall Street, 22nd Floor New York, NY 10005, USA. [5]Department of Epidemiology, University of Pittsburgh, 127 Parran Hall, Pittsburgh, PA 15261, USA. [6]Brain Resource International Database, Brain Resource Ltd, Sydney, Australia and San Francisco, USA. [7]Department of Psychiatry and Behavioral Sciences, University of Miami Miller School of Medicine, Miami FL 33136, USA.

[8]Department of Psychiatry and Behavioral Sciences, Stanford University School of Medicine, Stanford, CA 94305, USA.

Authors' contributions

LMW made substantial contributions to conception and design of the trial, provided important intellectual content to the manuscript, was substantially involved in drafting and revising the manuscript, and gave final approval of the submitted version of the manuscript. AJR made substantial contributions to conception and design of the trial, important intellectual content to the manuscript, was substantially involved in drafting and revising the manuscript, and gave final approval of the submitted version of the manuscript. SHK made substantial contributions to conception of the trial, was substantially involved in drafting and revising the manuscript, and gave final approval of the submitted version of the manuscript. SW made substantial contributions to the methods and statistical analysis for the trial, was substantially involved in drafting and revising the manuscript, and gave final approval of the submitted version of the manuscript. NC made substantial contributions to the methods and statistical analysis for the trial, was substantially involved in drafting and revising the manuscript, and gave final approval of the submitted version of the manuscript. CBN made substantial contributions to design of the trial, was substantially involved in drafting and revising the manuscript, and gave final approval of the submitted version of the manuscript. AFS made substantial contributions to design of the trial, was substantially involved in drafting and revising the manuscript, and gave final approval of the submitted version of the manuscript. EG conceptualized the design of the study, was substantially involved in drafting and revising the manuscript, and gave final approval of the submitted version of the manuscript.

Authors' Information

LMW is founding Chair of the BRAINnet Foundation, a non-profit global consortium for integrative neuroscience (headquartered in California, USA), and Professor in Cognitive Neuropsychiatry at Sydney Medical School. AJR is Vice Dean, Clinical Sciences at Duke-National University of Singapore Graduate medical School. Before this, he was Professor and Vice Chair in Clinical Sciences at Southwestern Medical Centre, US. AJR is an alumnus of Princeton University (biochemistry) and Doctor of Medicine from Columbia College of Physicians, with medical training at Northwestern University and psychiatry training at University of Pennsylvania. He has directed the largest treatment trial of depression ever conducted: the STAR*D trial http://www.star-d.org. AJR is Chair of the iSPOT-D Publication Committee. SHK is Research Director for the American Foundation for Suicide Prevention, and Director on the board of the BRAINnet Foundation. He was the first Director of the Neuroscience Research Branch at the NIMH where he was responsible for initiating new research programs including Human Brain Imaging. SHK was founder of the International Neuroinformatics Coordinating Facility. SW is a Professor in the Department of Epidemiology and Co-Director of the Epidemiology Data Center, University of Pittsburgh. His secondary appointment is in Psychiatry. He is an Associate Dean for Research in the Graduate School of Public Health. CBN is Leonard M. Miller Professor and Chairman of the Department of Psychiatry and Behavioral Sciences, University of Miami. Before this, he built the Department of Psychiatry at Emory University School of Medicine in Atlanta into one of the top ten departments in the United States, as Reunette W. Harris Professor and Chairman of the Department of Psychiatry and Behavioral Sciences. AFS is the Chair of Stanford School of Medicine - Psychiatry and Behavioral Sciences. EG was founding Director of the Brain Dynamics Centre (BDC), Sydney Medical School. He has founded the first standardized international database on the human brain.

Competing interests

LMW has received consulting fees and stock options in Brain Resource Ltd, and is a stock holder in Brain Resource Ltd. She has received Advisory Board fees from Pfizer. AJR has received consulting fees from Advanced Neuromodulation Systems, AstraZeneca, Best Practice Project Management, Brain Resource Ltd, Bristol-Myers Squibb/Otsuka, Cyberonics, Gerson Lehrman Group, Jazz Pharmaceuticals, Magellan Health Services, Merck and Company, Neuronetics, Novartis Pharmaceuticals, Ono Pharmaceuticals, Organon, Otsuka Pharmaceuticals, Pamlab, Transcept Pharmaceuticals, Urban Institute, Wyeth Ayerst and University of Michigan. AJR has received fees for consulting and as speaker for consulting fees and honoraria from Cyberonics, Forest Pharmaceuticals, GlaxoSmithKline and Pfizer. He is a stock holder in Pfizer. AJR has received a stipend from Society of Biological Psychiatry (as Treasurer). He has author royalties from Guilford Publications, Healthcare Technology Systems and University of Texas Southwestern Medical Center. SHK has received income as Research Director, from the American Foundation for Suicide Prevention. He has received fees and stock options as consultant, from Brain Resource Ltd. SHK has received consultant fees from Biomedical Synergy. SRW has received fees as consultant from Cyberonic Inc, ImaRx Therapeutics Inc, Bristol-Myers Squibb Company, Organon, Case-Western University and Singapore Clinical Research Institute. NJC has received income and stock options as senior statistician employee with Brain Resource Ltd. CBN has received a Scientific Advisory Board/Board of Directors fee from AstraZeneca, PharmaNeuroboost, Forest Laboratories, NARSAD, Quintiles, Janssen/Ortho-McNeil, Mt. Cook Pharma Inc, George West Mental Health Foundation and NovaDel Pharma. He holds stock in Corcept, CeNeRx, ReVax, PharmaNeuroboost, Novadel Pharma. CBN has patents for Methods and devices for the transdermal delivery of lithium (US 6,375,990 BI) and Method to estimate serotonin and norepinephrine transporter occupancy after drug treatment using patient or animal serum (provisional filing April, 2001). AFS has received fees as consultant from Brain Cells, CeNeRxm, CNS Response, Corcept, Glaxo-Smith Kline, Merck and Company, Neuronectics, PharmaNeuroBoost, Sanofi-Aventis and Takeda. He is a stock holder in Amnestix, Brain Cells, CeNeRx, Corpect, Forest, Merck and Company, Neurocrine, Pfizer, PharmaNeuroBoost, Somaxon and Synosis. AFS is cofounder of Corcept.
EG is founder and receives income as Chief Executive Officer and Chairman for Brain Resource Ltd. He has stock options in Brain Resource Ltd.

Received: 10 August 2010 Accepted: 5 January 2011
Published: 5 January 2011

References

1. Greenberg PE, Stiglin LE, Finkelstein SN, Berndt ER: **The economic burden of depression in 1990.** *J Clin Psychiatry* 1993, **54**:405-418.
2. Murray CJ, Lopez AD: **Evidence-based health policy–lessons from the Global Burden of Disease Study.** *Science* 1996, **274**:740-743.
3. Kessler RC, Berglund P, Demler O, Jin R, Koretz D, Merikangas KR, Rush AJ, Walters EE, Wang PS, National Comorbidity Survey Replication: **The epidemiology of major depressive disorder: results from the National Comorbidity Survey Replication (NCS-R).** *JAMA* 2003, **289**:3095-3105.
4. Simon GE, Von Korff M, Barlow W: **Health care costs of primary care patients with recognized depression.** *Arch Gen Psychiatry* 1995, **52**:850.
5. Frank E, Karp JF, Rush AJ: **Efficacy of treatments for major depression.** *Psychopharmacol Bull* 1993, **29**:457-475.
6. Depression Guideline Panel: *Clinical practice guideline. Number 5. Depression in primary care: volume 2. Treatment of major depression* Rockville, MD: U.S. Department of Health and Human Services, Public Health Service, Agency for Health Care Policy and Research; 1993, AHCPR Publication No. 93-0551.
7. Thase ME, Rush AJ: **Treatment-resistant depression.** In *Psychopharmacology: fourth generation of progress.* Edited by: Bloom FE, Kupfer DJ. New York: Raven Press; 1995:1081-1097.
8. American Psychiatric Association: **Practice guideline for major depressive disorder in adults.** *Am J Psychiatry* 1993, **150(Suppl 4)**:1-2.
9. Rosenbaum JF, Fava M, Nierenberg AA, Sachs GS: **Treatment-resistant mood disorders.** In *Treatments of psychiatric disorders.* 3 edition. Edited by: Gabbard GO. Washington, DC: American Psychiatric Press, Inc; 2001:1307-1384.
10. Fava M, Davidson KG: **Definition and epidemiology of treatment-resistant depression.** *Psychiatr Clin North Am* 1996, **19**:179-200.
11. Frank E, Prien R, Jarrett RB, Keller MB, Kupfer DJ, Lavori PW, Rush AJ, Weissman MM: **Conceptualization and rationale for consensus definitions of terms in major depressive disorder. Remission, recovery, relapse and recurrence.** *Arch Gen Psychiatry* 1991, **48**:851-855.
12. Trivedi MH, Rush AJ, Wisniewski SR, Nierenberg AA, Warden D, Ritz L, Norquist G, Howland RH, Lebowitz B, McGrath PJ, Shores-Wilson K, Biggs MM, Balasubramani GK, Fava M, STAR*D Study Team: **Evaluation of outcomes with citalopram for depression using measurement-based care in STAR*D: implications for clinical practice.** *Am J Psychiatry* 2006, **163**:28-40.
13. Miller IW, Keitner GI, Schatzberg AJ, Klein DN, Thase ME, Rush AJ, Markowitz JC, Schlager DS, Kornstein SG, Davis SM, Harrison WM, Keller MB:

The treatment of chronic depression, Part 3: psychosocial functioning before and after treatment with sertraline or imipramine. *J Clin Psychiatry* 1998, **59**:608-619.
14. Judd LL, Paulus MP, Wells KB: **Socioeconomic burden of subsyndromal depressive symptoms and major depression in a sample of the general population.** *Am J Psychiatry* 1996, **153**:1411-1416.
15. Judd LL: **The clinical course of unipolar major depressive disorders.** *Arch Gen Psychiatry* 1997, **54**:989-991.
16. Rush AJ, Trivedi MH: **Treating depression to remission.** *Psychiatr Ann* 1995, **25**:704-705.
17. Van Londen L, Molenaar RP, Goekoop JG, Zwinderman AH, Rooijmans HG: **Three- to 5-year prospective follow-up of outcome in major depression.** *Psychol Med* 1998, **28**:731-735.
18. Sackeim HA, Prudic J, Devanand DP, Nobler MS, Lisanby SH, Peyser S, Fitzsimons L, Moody BJ, Clark J: **A prospective, randomized, double-blind comparison of bilateral and right unilateral electroconvulsive therapy at different stimulus intensities.** *Arch Gen Psychiatry* 2000, **57**:425-434.
19. Rush AJ, Fava M, Wisniewski SR, Lavori PW, Trivedi MH, Sackeim HA, Thase ME, Nierenberg AA, Quitkin FM, Kashner TM, Kupfer DJ, Rosenbaum JF, Alpert J, Stewart JW, McGrath PJ, Biggs MM, Shores-Wilson K, Lebowitz BD, Ritz L, Niederehe G, STAR*D Investigators Group: for the STAR*D Investigators Group: **Sequenced Treatment Alternatives to Relieve Depression (STAR*D): Rationale and Design.** *Control Clin Trials* 2004, **25**:116-142.
20. Kraemer HC, Wilson GT, Fairburn CG, Agras WS: **Mediators and moderators of treatment effects in randomized clinical trials.** *Arch Gen Psychiatry* 2002, **59**:877-883.
21. Gordon E, Williams LML: **Personalized Medicine and Integrative Neuroscience: towards consensus markers for disorders of brain health.** In *Personalized Medicine, Healthcare and Integrative Neuroscience.* Edited by: Gordon E, Koslow SH. New York: Oxford University Press; 2010.
22. Rush AJ: **STAR*D: What have we learned?** *Am J Psychiatry* 2007, **164**:201-204.
23. Joyce PR, Paykerl ES: **Predictors of drug response in depression.** *Arch Gen Psychiatry* 1989, **46(1)**:88-99.
24. Hennings JM, Owashi T, Binder EB, Horstmann S, Menke A, Kloiber S, Dose T, Wollweber B, Spieler D, Messer T, Lutz R, Künzel H, Bierner T, Pollmächer T, Pfister H, Nickel T, Sonntag A, Uhr M, Ising M, Holsboer F, Lucae S: **Clinical characteristics and treatment outcome in a representative sample of depressed inpatients - Findings from the Munich Antidepressant Response Signature (MARS) project.** *J Psychiatr Res* 2009, **43**:215-229.
25. Rush AJ, Wisniewski SR, Warden D, Luther JF, Davis LL, Fava M, Nierenberg AA, Trivedi MH: **Selecting Among Second-Step Antidepressant Medication Monotherapies, Predictive Value of Clinical, Demographic, or First-Step Treatment Features.** *Arch Gen Psychiatry* 2008, 65**(8)**:870-880.
26. March JS, Silva SG, Compton S, Shaprio M, Califf R, Krishman R: **The case for practical clinical trials in psychiatry.** *Am J Psychiatry* 2005, 162:836-846.
27. Tunis SR, Stryer DB, Clancy CM: **Practical trials: increasing the value of clinical research for decision making in clinical and health policy.** *JAMA* 2003, **290**:1624-1632.
28. Williams LM, Gatt JM, Hatch A, Palmer DM, Nagy M, Rennie C, Cooper NJ, Morris C, Grieve S, Dobson-Stone C, Schofield P, Clark CR, Gordon E, Arns M, Paul RH: **The INTEGRATE Model of emotion, thinking and self regulation: an application to the 'paradox of aging'.** *J Integr Neurosci* 2008, **7(3)**:367-404.
29. Gordon E: **Integrative Neuroscience.** *Neuropsychopharmacology* 2003, **28(Suppl 1)**:S2-8.
30. Gordon E, Barnett KJ, Cooper N, Tran N, Williams LM: **An 'integrative neuroscience' platform: application to profiles of negativity and positivity.** *J Integr Neurosci* 2008, **7(3)**:345-366.
31. Sheehan DV, Lecrubier Y, Harnett-Sheehan K, Janavs J, Weiller E, Keskiner A, Schinka J, Knapp E, Sheehan MF, Dunbar GC: **Reliability and Validity of the MINI International Neuropsychiatric Interview (M.I.N.I.): According to the SCID-P.** *European Psychiatry* 1997, **12**:232-241.
32. Sheehan DV, Lecrubier Y, Sheehan KH, Amorim P, Janavs J, Weiller E, Hergueta T, Baker R, Dunbar GC: **The Mini-International Neuropsychiatric Interview (M.I.N.I.): The development and validation of a structured diagnostic psychiatric interview for DSM-IV and ICD-10.** *J Clin Psychiatry* 1998, **59(Suppl 20)**:22-33.
33. Hamilton M: **A rating scale for depression.** *J Neurol Neurosurg Psychiatry* 1960, **23**:56-61.
34. Rush AJ, Carmody T, Reimitz PE: **The Inventory of Depressive Symptomatology (IDS): Clinician (IDS-C) and self-report (IDS-SR) ratings of depressive symptoms.** *International Journal of Methods Psychiatric Research* 2000, **9**:45-59.
35. Rush AJ, Trivedi MH, Ibrahim HM, Carmody TJ, Arnow B, Klein DN, Markowitz JC, Ninan PT, Kornstein S, Manber R, Thase ME, Kocsis JH, Keller MB: **The 16-item Quick Inventory of Depressive Symptomatology (QIDS) Clinician Rating (QIDS-C) and Self-Report (QIDS-SR): a psychometric evaluation in patients with chronic major depression.** *Biol Psychiatry* 2003, **54**:573-583.
36. Trivedi MH, Rush AJ, Ibrahim HM, Carmody TJ, Biggs MM, Suppes T, Crismon ML, Shores-Wilson K, Toprac MG, Dennehy EB, Witte B, Kashner TM: **The Inventory of Depressive Symptomatology, clinician rating (IDS-C) and self-report (IDS-SR), and the Quick Inventory of Depressive Symptomatology, clinician rating (QIDS-C) and self-report (QIDS-SR) in public sector patients with mood disorders: A psychometric evaluation.** *Psychol Med* 2004, **34**:73-82.
37. Binder EB, Holsboer F: **Pharmacogenomics and antidepressant drugs.** *Ann Med* 2006, **38**:82-94.
38. Choi MJ, Kang RH, Lim SW, Oh KS, Lee MS: **Brain-derived neurotrophic factor gene polymorphism (Val66Met) and citalopram response in major depressive disorder.** *Brain Res* 2006, **1118**:176-182.
39. Paddock S, Laje G, Charney D, Rush AJ, Wilson AF, Sorant AJ, Lipsky R, Wisniewski SR, Manji H, McMahon FJ: **Association of GRIK4 with Outcome of Antidepressant Treatment in the STAR*D Cohort.** *Am J Psychiatry* 2007, **164**:1181-1188.
40. Binder EB, Salyakina D, Lichtner P, Wochnik GM, Ising M, Pütz B, Papiol S, Seaman S, Lucae S, Kohli MA, Nickel T, Künzel HE, Fuchs B, Majer M, Pfennig A, Kern N, Brunner J, Modell S, Baghai T, Deiml T, Zill P, Bondy B, Rupprecht R, Messer T, Köhnlein O, Dabitz H, Brückl T, Müller N, Pfister H, Lieb R, Mueller JC, Lõhmussaar E, Strom TM, Bettecken T, Meitinger T, Uhr M, Rein T, Holsboer F, Muller-Myhsok B: **Polymorphisms in FKBP5 are associated with increased recurrence of depressive episodes and rapid response to antidepressant treatment.** *Nat Genet* 2004, **36**:1319-1325.
41. Nemeroff CB: **Recent findings in the pathophysiology of depression.** *Am J Psychiatry* 2008, **6**:3-14.
42. Zanardi R, Benedetti F, Di Bella D, Catalano M, Smeraldi E: **Efficacy of paroxetine in depression is influenced by a functional polymorphism within the promoter of the serotonin transporter gene.** *J Clin Psychopharmacol* 2000, **20**:105-107.
43. Tsai SJ, Cheng CY, Yu YW, Chen TJ, Hong CJ: **Association study of a brain-derived neurotrophicfactor genetic polymorphism and major depressive disorders, symptomatology, and antidepressant response.** *Am J Med Genet B Neuropsychiatr Genet* 2003, **123**:19-22.
44. Villafuerte SM, Vallabhaneni K, Sliwerska E, McMahon FJ, Young EA, Burmeister M: **SSRI response in depression may be influenced by SNPs in HTR1B and HTR1A.** *Psychiatr Genet* 2009, **19(6)**:281-291.
45. Gatt JM, Nemeroff CB, Dobson-Stone C, Paul RH, Bryant RA, Schofield PR, Gordon E, Kemp AH, Williams LM: **Interactions between BDNF Val66Met polymorphism and early life stress predict brain and arousal pathways to syndromal depression and anxiety.** *Mol Psychiatry* 2009, **14(7)**:681-695.
46. Lee BT, Ham BJ: **Serotonergic genes and amygdala activity in response to negative affective facial stimuli in Korean women.** *Genes Brain Behav* 2008, **7**:899-905.
47. Williams LM, Gatt JM, Schofield PR, Olivieri G, Peduto A, Gordon E: **'Negativity bias' in risk for depression and anxiety: brain-body fear circuitry correlates, 5-HTT-LPR and early life stress.** *Neuroimage* 2009, **47(3)**:804-814.
48. Wisniewski SR, Rush AJ, Balasubramani GK, Trivedi MH, Nierenberg AA, for the STAR*D Investigators: **Self-rated global measure of the frequency, intensity, and burden of side effects.** *J Psychiatr Pract* 2006, 12**(2)**:71-79.
49. World Health Organization Group: **Development of the World Health Organization WHOQOL-BREF quality of life assessment.** *Psychol Med* 1998, **28**:551-558.
50. Goldman HH, Skodol AE, Lave TR: **Revising axis V for DSM-IV: a review of measures of social functioning.** *Am J Psychiatry* 1992, **149**:1148-1156.
51. Gross JJ, John OP: **Individual differences in two emotion regulation processes: Implications for affect, relationships and well-being.** *J Pers Soc Psychol* 2003, **85**:348-362.
52. Lovibond PF, Lovibond SH: *Manual for the Depression Anxiety Stress Scales.* 2 edition. Sydney: Psychological Foundation; 1995.

53. Lovibond PF: **Long-term stability of Depression, Anxiety and Stress syndromes.** *J Abnorm Psychol* 1998, **107**:520-526.
54. Cohen RA, Grieve S, Hoth KF, Paul RH, Sweet L, Tate D, Gunstad J, Stroud L, McCaffery J, Hitsman B, Niaura R, Clark CR, McFarlane A, Bryant R, Gordon E, Williams LM: **Early life stress and morphometry of the adult anterior cingulate cortex and caudate nuclei.** *Biol Psychiatry* 2006, **59(10)**:975-982.
55. Williams LM, Mathersul D, Palmer DM, Gur RC, Gur RE, Gordon E: **Explicit identification and implicit recognition of facial emotions: I. Age effects in males and females across 10 decades.** *J Clin Exp Neuropsychol* 2009, **31(3)**:257-277.
56. Clark CR, Paul RH, Williams LM, Arns M, Fallahpour K, Handmer C, Gordon E: **Standardized assessment of cognitive functioning during development and aging using an automated touchscreen battery.** *Arch Clin Neuropsychol* 2006, **21(5)**:449-467.
57. Gunstad J, Paul RH, Brickman AM, Cohen RA, Arns M, Roe D, Lawrence JJ, Gordon E: **Patterns of cognitive performance in middle-aged and older adults: A cluster analytic examination.** *J Geriatr Psychiatry Neurol* 2006, **19(2)**:59-64.
58. Williams LM, Simms E, Clark CR, Paul RH, Rowe D, Gordon E: **The test-retest reliability of a standardized neurocognitive and neurophysiological test battery: "neuromarker".** *Int J Neurosci* 2005, **115(12)**:1605-1630.
59. Paul RH, Lawrence J, Williams LM, Richard CC, Cooper N, Gordon E: **Preliminary validity of "integneuro": a new computerized battery of neurocognitive tests.** *Int J Neurosci* 2005, **115(11)**:1549-1567.
60. Paul RH, Gunstad J, Cooper N, Williams LM, Clark CR, Cohen RA, Lawrence JJ, Gordon E: **Cross-cultural assessment of neuropsychological performance and electrical brain function measures: additional validation of an international brain database.** *Int J Neurosci* 2007, **117(4)**:549-568.
61. Brickman AM, Zimmerman ME, Paul RH, Grieve SM, Tate DF, Cohen RA, Williams LM, Clark CR, Gordon E: **Regional white matter and neuropsychological functioning across the adult lifespan.** *Biol Psychiatry* 2006, **60(5)**:444-453.
62. Zimmerman ME, Brickman AM, Paul RH, Grieve SM, Tate DF, Gunstad J, Cohen RA, Aloia MS, Williams LM, Clark CR, Whitford TJ, Gordon E: **The relationship between frontal gray matter volume and cognition varies across the healthy adult lifespan.** *Am J Geriatr Psychiatry* 2006, **14(10)**:823-833.
63. Grieve SM, Williams LM, Paul RH, Clark CR, Gordon E: **Cognitive aging, executive function, and fractional anisotropy: a diffusion tensor MR imaging study.** *AJNR Am J Neurorad* 2007, **28(2)**:226-235.
64. Hatch A, Madden S, Kohn MR, Clarke S, Touyz S, Gordon E, Williams LM: **In first presentation adolescent anorexia nervosa, do cognitive markers of underweight status change with weight gain following a refeeding intervention?** *Int J Eat Disord* 2010, **43(4)**:295-306.
65. Liddell BJ, Paul RH, Arns M, Gordon N, Kukla M, Rowe D, Cooper N, Moyle J, Williams LM: **Rates of Decline Distinguish Alzheimer's Disease and Mild Cognitive Impairment relative to Normal Aging: Integrating Cognition and Brain Function.** *J Integr Neurosci* 2007, **6(1)**:141-174.
66. Silverstein SM, Jaeger J, Lepore D, Wilkniss SM, Savitz A, Malinovsky I, Hawthorne D, Raines S, Carson S, Marcello S, Zukin SR, Furlong S, Dent G: **A comparative study of the MATRICS and IntegNeuro cognitive assessment batteries.** *J Clin Exp Neuropsychol* 2010, **7**:1-16.
67. Williams LM, Whitford TJ, Flynn G, Wong W, Liddell BJ, Silverstein S, Galletly C, Harris AW, Gordon E: **General and social cognition in first episode schizophrenia: Identification of separable factors and prediction of functional outcome using the IntegNeuro test battery.** *Schizophr Res* 2008, **99**:182-191.
68. Williams LM, Hermens DF, Thein T, Clark CR, Cooper NJ, Clarke SD, Lamb C, Gordon E, Kohn MR: **Using brain-based cognitive measures to support clinical decisions in ADHD.** *Pediatr Neurol* 2010, **42**:118-126.
69. Wong KKH, Grunstein RR, Bartlerr DJ, Gordon E: **Brain function in obstructive sleep apnea: results from the Brain Resource International Database.** *J Integr Neurosci* 2006, **5(1)**:111-121.
70. Davidson RJ: **Anterior electrophysiological asymmetries, emotion, and depression: Conceptual and methodological conundrums.** *Psychophysiol* 1998, **35**:607-614.
71. Allen JJB, Urry HL, Hitt SK, Coan JA: **Stability of Resting Frontal EEG Asymmetry Across Different Clinical States of Depression.** *Psychophysiology* 2004, **41**:269-280.
72. Kemp AH, Hopkinson PJ, Hermens DF, Rowe DL, Sumich AL, Clark CR, Drinkenburg W, Abdi N, Penrose R, McFarlane A, Boyce P, Gordon E, Williams LM: **Fronto-temporal alterations within the first 200 ms during an attentional task distinguish major depression, non-clinical participants with depressed mood and healthy controls: A potential biomarker?** *Hum Brain Mapp* 2009, **30(2)**:602-614.
73. Task Force of the European Society of Cardiology the North American Society of Pacing Electrophysiology: **Heart rate variability: standards of measurement, physiological interpretation and clinical use.** *Circulation* 1996, **93**:1043-1065.
74. *Brain Resource Data Quality Control Manual* Ultimo: Brain Resource Ltd; 2010.
75. Kobak KA, Williams JB, Engelhardt N: **A comparison of face-to-face and remote assessment of inter-rater reliability on the Hamilton Depression Rating Scale via videoconferencing.** *Psychiatry Res* 2008, **158(1)**:99-103.

doi:10.1186/1745-6215-12-4
Cite this article as: Williams *et al.*: International Study to Predict Optimized Treatment for Depression (iSPOT-D), a randomized clinical trial: rationale and protocol. *Trials* 2011 **12**:4.

Submit your next manuscript to BioMed Central and take full advantage of:

- **Convenient online submission**
- **Thorough peer review**
- **No space constraints or color figure charges**
- **Immediate publication on acceptance**
- **Inclusion in PubMed, CAS, Scopus and Google Scholar**
- **Research which is freely available for redistribution**

Submit your manuscript at
www.biomedcentral.com/submit

BioMed Central
